# A Security-Enhanced Certificateless Aggregate Authentication Protocol with Revocation for Wireless Medical Sensor Networks

**DOI:** 10.3390/s26072106

**Published:** 2026-03-28

**Authors:** Quan Fan, Yimin Wang, Xiang Li

**Affiliations:** 1 School of Information and Artificial Intelligence, Anhui Agricultural University, Hefei 230036, China; qf@stu.ahau.edu.cn (Q.F.); lixiang000@stu.ahau.edu.cn (X.L.); 2 Anhui Provincial Key Laboratory of Industrial Intelligent Data Security, Anhui Normal University, Wuhu 240002, China

**Keywords:** wireless medical sensor networks, certificateless aggregate signature (CLAS), RSA accumulator, attack, zero-knowledge membership proof, revocation, unlinkability

## Abstract

Wireless medical sensor networks (WMSNs) enable continuous patient monitoring by transmitting sensitive physiological data over open wireless links. Given the resource-constrained nature and large-scale deployment of such networks, authentication mechanisms must be both lightweight and privacy-preserving. Moreover, due to the frequent turnover of patients and devices in hospital environments, timely member revocation is crucial to prevent discharged or compromised entities from injecting forged reports that could mislead medical diagnosis. Although existing pairing-free certificateless aggregate authentication schemes are efficient, they often suffer from critical security and privacy vulnerabilities. Recently, an efficient certificateless authentication scheme with revocation has been proposed. However, our analysis reveals that the scheme presents the following security vulnerabilities: (i) member witnesses can be recovered from public information, (ii) revocation checks can be bypassed via identity grafting attack, and (iii) user identities can be linked due to the long-term use of static pseudonyms. To address these issues, we propose a security-enhanced certificateless aggregate authentication protocol with revocation for WMSNs. Our design enforces strong identity–membership binding to resist grafting attacks, employs a non-interactive zero-knowledge membership proof to preserve witness secrecy, and adopts dynamic pseudonym rotation to achieve unlinkability. We provide formal security proofs and comprehensive performance comparisons. The results indicate that, at the same security level, our protocol achieves more efficient signature verification while maintaining communication overhead comparable to existing schemes. In addition, the overhead introduced by our revocation mechanism remains constant, making it well suited for large-scale WMSNs deployments with frequent membership changes.

## 1. Introduction

Wireless medical sensor networks (WMSNs) are a transformative healthcare-oriented IoT paradigm that integrates wearable or implantable sensors to establish a comprehensive monitoring ecosystem [1]. The continuous collection and reporting of critical physiological signals, including heart rate, blood pressure, and blood glucose levels, has been demonstrated to facilitate chronic disease management, rehabilitation tracking, and real-time in-hospital monitoring [2]. However, such networks are inherently exposed to eavesdroppable and interference-prone wireless environments, and the system attack surface is inevitably expanded, exposing patients to the risks of privacy leakage and data tampering.

Authentication mechanisms for WMSNs must achieve a high level of security while also accommodating strict resource constraints [3]. On the one hand, the integrity and authenticity of medical data are non-negotiable; once sensitive medical data are tampered with or forged, this may directly mislead clinical decisions and even endanger patients’ lives. On the other hand, sensor nodes are typically battery-powered and have limited computation, storage, and bandwidth. Consequently, traditional public key infrastructure (PKI) becomes too heavyweight due to costly certificate management [4], while identity-based cryptography (IBC) introduces key-escrow risks [5]. Certificateless public key cryptography (CL-PKC) does not inherit intrinsic escrow concerns while eliminating certificates, and thus emerges as a balanced solution. In addition, to alleviate network congestion caused by concurrent reporting from massive nodes, researchers have proposed various “aggregation” mechanisms, which can be mainly classified into data aggregation and signature aggregation. Data aggregation aims to statistically or computationally fuse multiple sensor readings to extract meaningful information while reducing data volume [6], but it usually needs to be processed at intermediate aggregators, which may enlarge the privacy exposure surface and introduce tampering or injection risks. In contrast, signature aggregation focuses on the cryptographic scalability of authentication. Certificateless aggregate signature (CLAS) schemes allow aggregators to compress multiple signatures into a single verifiable object for batch verification, reducing the authentication communication and verification overhead [7]. However, early CLAS constructions mostly relied on expensive bilinear pairing operations, and recent research has shifted towards the design of more lightweight pairing-free aggregatable signatures.

Despite these advances, a key gap remains when deploying them to real medical environments. Hospital settings are highly dynamic: frequent events such as patient discharge, device replacement, sensor loss, or temporary device lending require the system to revoke membership promptly; otherwise, compromised or misplaced devices may continue to authenticate with still-valid credentials and inject forged data. Meanwhile, the long-term use of static pseudonyms may render messages linkable, enabling patient privacy to be continuously tracked across sessions and over time. Therefore, authentication mechanisms for practical WMSNs must not only be lightweight and efficient but also satisfy the engineering requirements of instant revocation and unlinkability.

Recently, Shen et al. [8] proposed a novel RSA-accumulator-based CLAS scheme that facilitates dynamic membership management without expensive bilinear pairings. Although their design demonstrates notable efficiency, our detailed cryptanalysis reveals several critical security flaws: (1) membership witnesses can be recovered from public information and cross-session observations; (2) insufficient binding between membership credentials and signing keys enables a revoked entity to graft another valid membership and bypass revocation checking; and (3) long-term static pseudonyms and public-key components render messages linkable, endangering patient location and behavioral privacy.

Motivated by these observations, we propose a security-enhanced revocable certificateless aggregate authentication protocol tailored for WMSNs, which patches these weaknesses while enabling more efficient authentication and revocation. Our main contributions are summarized as follows.

1. We revisit Shen et al.’s [8] scheme, identify its logical flaws in membership-witness protection and identity-binding mechanisms, and provide concrete attack paths.
2. We propose a security-enhanced certificateless aggregate authentication protocol. We introduce a strong identity–membership binding mechanism to prevent grafting attacks, employ a zero-knowledge membership proof to protect witness secrecy, and adopt dynamic pseudonym rotation to achieve enhanced privacy protection.
3. We provide formal security proofs and verification for the proposed protocol, and conduct systematic performance analysis and comparative evaluation in terms of computation, communication, and revocation overhead. The results demonstrate that our protocol achieves stronger security guarantees while maintaining favorable efficiency, making it more suitable for resource-constrained and dynamic WMSNs.

The remainder of this paper is organized as follows. Section 2 reviews related work. Section 3 introduces preliminaries and the system model. Section 4 and Section 5 review Shen et al.’s [8] scheme and present the corresponding cryptanalysis and concrete attack scenarios. Section 6 details our proposed protocol. Section 7 and Section 8 provide the security analysis and performance evaluation. Finally, Section 9 and Section 10 discuss the feasibility, limitations and future work of the paper, and give the final conclusion.

## 2. Related Work

The evolution of authentication protocols in WMSNs is largely constrained by the need to balance stringent security requirements with the limited resources of sensor nodes [9]. PKI-based solutions rely on digital certificates, yet certificate issuance, storage, and validation introduce additional management and communication overheads [10]. Identity-based cryptography (IBC/IBS) derives public keys from identities to simplify certificate management [11]; however, since user private keys are generated by a key generation center (KGC), IBC inherently suffers from the key-escrow problem. Although pairing-free IBS designs can reduce computation in sensor networks, they do not fundamentally eliminate the risks associated with escrow risks [12].

To balance the certificate-management burden and the key-escrow risk, Al-Riyami and Paterson introduced certificateless public key cryptography (CL-PKC), while the original construction was later shown to be insecure against Type-II adversaries [13]. Following CL-PKC, many schemes employed bilinear pairings to realize authentication, anonymity, and aggregation [14,15,16]. For instance, Meher et al. proposed a certificateless anonymous mutual-authentication protocol for WBANs and adopted a hybrid design combining DLP, ECDLP, and bilinear pairings to ensure security [16]. Nevertheless, pairing operations are expensive and thus remain unsuitable for resource-constrained WMSNs. In contrast, recent studies have increasingly explored pairing-free (ECC-based) certificateless authentication and certificateless aggregate signature (CLAS) designs. In the domain of healthcare WMSNs, Gayathri et al. proposed an efficient pairing-free CLAS scheme [17]. Liu et al. later proved that this scheme fails against Type-I and Type-II adversaries and presented an improved construction [18]. Subsequently, Qiao et al. analyzed Liu et al.’s scheme, demonstrated that it remains insecure against Type-II adversaries, and further proposed a new pairing-free CLAS scheme [19]. However, Yan et al. demonstrated that Qiao et al.’s scheme is still vulnerable to Type-I adversaries [20]. In the field of certificateless conditional privacy-preserving authentication for highly dynamic networks, Zhu et al. devised a security-enhanced scheme [21]. Yang et al. demonstrated the scheme’s vulnerability to Type-I and Type-III adversaries and proposed an improved design [22]; subsequently, Wu et al. subsequently revealed the vulnerability of Yang et al.’s improved scheme and presented a new certificateless aggregate signature construction to enhance security [23]. Overall, many pairing-free certificateless proposals repeatedly undergo a cycle of proposal, cryptanalysis, and patching under standard certificateless threat models. Moreover, the majority of these pairing-free designs do not take member revocation into consideration, a factor which serves to limit their suitability for the highly dynamic nature of practical WMSNs.

It is evident that, in order to address the limitations in authentication that are a consequence of dynamic membership changes, a number of revocation strategies have been integrated into certificateless settings. The most straightforward approach is based on certificate revocation lists (CRLs) or blacklists. The schemes devised by Zhang et al. and Guo et al. employ CRLs or blacklists [24,25]. However, Zhang et al.’s revocation check mainly targets the validity of specific signatures rather than directly revoking the signer’s membership, which limits its effectiveness against compromised nodes. Furthermore, Guo et al.’s scheme was recently reported to be vulnerable to Type-I adversaries [26]. Furthermore, the proliferation of such lists is directly proportional to the number of revoked users, resulting in a substantial augmentation of the storage and broadcast burden. In order to reduce the financial burden associated with CRL maintenance costs, Zhou et al. adopted the utilization of periodic time keys [27]. However, the revocation process may be subject to delays, owing to the implementation of discrete update intervals. Subsequently, Li et al. proposed an immediate-update revocation mechanism [28], and Wang et al. designed a revocation method by updating an area-related private key [29]. However, these approaches typically require the recomputation and distribution of fresh credentials to legitimate users via secure point-to-point delivery, resulting in limited update efficiency. Although Liang et al.’s polynomial-broadcast revocation update [30] circumvents point-to-point communication, the broadcast polynomial must embed coefficients for all current legitimate members, which may still incur substantial computation or communication costs during updates. Consequently, this may impede scalability in WMSNs.

In order to address the issues of scalability, the use of cryptographic accumulators as a compact alternative has been explored [31]. Representative accumulator models include those based on Merkle hash trees, bilinear pairings, and the strong RSA assumption. Camacho et al. [32] proposed a Merkle-hash-tree-based scheme that defines the root as the accumulator value. However, the membership proof size is O(logN). This logarithmic growth significantly increases communication costs as the network scales. Camenisch et al. [33] proposed a pairing-based accumulator framework. This framework requires a predefined upper bound *q* on the number of members, features O(q)-size public parameters, and incurs high computational costs due to pairing operations. As a result, it is unsuitable for large-scale, resource-constrained networks. In contrast, RSA cryptographic accumulators provide O(1)-size public parameters and O(1)-size membership witnesses, while supporting unbounded dynamic additions and deletions [34], which better matches the lightweight computation and efficient communication requirements of dynamic environments such as WMSNs. In recent work, Shen et al. proposed a certificateless authentication scheme based on an RSA accumulator [8]. This scheme employs an accumulator value and a membership witness to facilitate dynamic management of membership states. It disseminates update information via broadcast, thereby achieving notable efficiency advantages. However, subsequent analysis indicates that the construction still leaves several security aspects open. This paper proposes a revocable certificateless aggregate authentication protocol that is both more secure and more efficient, together with a more rigorous security proof and performance evaluation.

## 3. Preliminaries

In this section, we provide a concise overview of the cryptographic primitives employed in our construction. The subsequent section will introduce the system and security models of the proposed certificateless authentication scheme. The primary notations employed in the present proposal are outlined in Table 1.

### 3.1. Cryptographic Preliminaries

#### 3.1.1. Elliptic Curve Cryptography

Elliptic curve cryptography (ECC) is based on group operations over an elliptic curve defined on a finite field, and is usually modeled as an additive cyclic group G. Let G be of order *q* with generator *P*. For any $x\in Z_{q}^{*}$, scalar multiplication is denoted by xP.

#### 3.1.2. RSA Accumulator

RSA accumulator is a cryptographic primitive that aggregates a large set of values into a constant-size digest. Let $X=\{x_{1},x_{2},\ldots ,x_{n}\}$ be a set of prime numbers. The accumulator value is computed as $Acc=g^{\prod_{i=1}^{n}x_{i}}\;\left(\mathrm{mod}\;N\right)$, where *N* is an RSA modulus and *g* is a generator. For a specific element $x_{k}\in X$, its membership witness $w_{k}$ is the accumulation of all other elements: $w_{k}=g^{\prod_{x_{j}\in X,j\neq k}x_{j}}\;\left(\mathrm{mod}\;N\right)$. The membership of $x_{k}$ can be efficiently verified by checking the equation $w_{k}^{x_{k}}\equiv Acc\;\left(\mathrm{mod}\;N\right)$.

#### 3.1.3. Non-Interactive Zero-Knowledge Proof

Non-interactive zero-knowledge (NIZK) proofs allow a prover to convince a verifier that a public statement holds with respect to a hidden witness, while revealing nothing about the witness and requiring no interaction. In this paper, we adopt a Fiat–Shamir-based NIZK membership proof [35], whose proof generation and verification typically follow a three-move structure as follows:

(1) Commitment: The prover generates a commitment based on fresh randomness.

(2) *Challenge:* The challenge is computed via a hash function over the statement and the commitment to bind them.

(3) *Response:* The prover computes a response from the witness, the randomness, and the challenge.

The construction of NIZK proofs typically requires to satisfy completeness, zero-knowledge, and soundness so that an honest prover can always generate an acceptable proof, no verifier can learn any additional secret information from the proof, and no malicious prover can convince the verifier of a false statement.

### 3.2. Security Assumptions

#### 3.2.1. ECDLP Assumption

The security of our ECC-based public-key components relies on the Elliptic Curve Discrete Logarithm Problem (ECDLP). Specifically, given a pair (P,Q) where Q=xP for an unknown $x\in Z_{q}^{*}$, no probabilistic polynomial-time (PPT) adversary can compute *x* with non-negligible probability.

#### 3.2.2. Strong RSA Assumption

The security of the RSA accumulator relies on the Strong RSA assumption. Given an RSA modulus *N* and a random element $z\in Z_{N}^{*}$, the Strong RSA assumption states that no probabilistic polynomial-time (PPT) adversary can find a pair (u,e) such that $u^{e}\equiv z\;\left(\mathrm{mod}\;N\right)$ with non-negligible probability, where *e* is a prime integer greater than 1.

#### 3.2.3. Integer Factorization Assumption

Let N=pq be an RSA modulus, where *p* and *q* are large primes. The integer factorization assumption states that for any probabilistic polynomial-time (PPT) adversary, the success probability of factoring *N* to recover *p* and *q* is negligible, which makes such factorization computationally infeasible.

### 3.3. System Model

**Figure 1:**
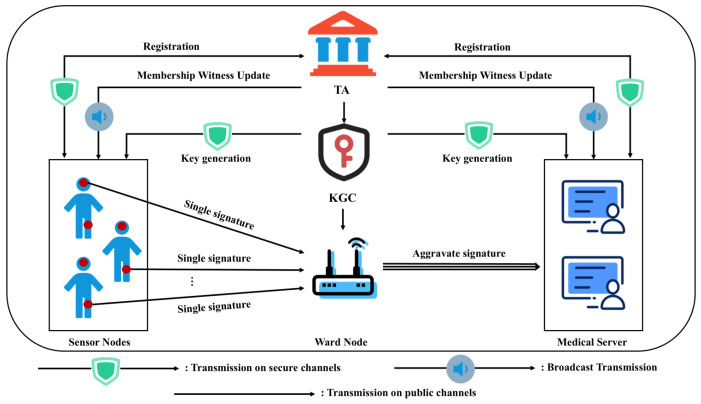
The system model of the proposed scheme.

Figure 1 depicts the system model of the proposed scheme, which involves five primary entities: Trusted Authority (TA), Key Generation Center (KGC), Sensor Node (SN), Ward Node (WN), and Medical Server (MS).

- **Trusted Authority (TA):** The TA is a fully trusted entity with significant computational capabilities. It is responsible for system initialization, generating RSA accumulator parameters, and generating pseudonyms for users. Additionally, the TA maintains the accumulator state and manages the batch joining and revocation of members by broadcasting update parameters.
- **Key Generation Center (KGC):** The KGC is a semi-trusted entity responsible for generating partial private keys. It cannot access the user’s full private key, thereby avoiding the key escrow problem.
- **Sensor Node (SN):** The SN is a resource-constrained device that is deployed on the patient. Its role is to collect physiological data such as heart rate and body temperature, sign the data, and transmit it to the Ward Node.
- **Ward Node (WN):** The WN functions as a gateway for a specific medical area, typically a hospital ward. It collects data transmitted by SNs within the area, verifies their membership proofs, and aggregates multiple valid signatures into a single one to reduce transmission bandwidth consumption. It then forwards the data to the Medical Server.
- **Medical Server (MS):** The MS is a back-end entity responsible for storing and processing medical data. It receives aggregated data from WNs, verifies the validity of the aggregate signatures, and provides authorized medical personnel with access to patients’ health statuses.

### 3.4. Security Model

In CLAS schemes, we adopt the standard security model and classify adversaries into five types according to their capabilities and resources, including the conventional Type-I to Type-III adversaries [21], as well as the attack-specific adversaries for witness recovery and identity grafting.

- **Type-I Adversary ($A_{I}$):** Models an external adversary capable of replacing public keys but lacking access to the master secret key.
- **Type-II Adversary ($A_{\mathrm{II}}$):** Models a malicious KGC possessing the master secret key but is restricted from replacing users’ public keys.
- **Type-III Adversary ($A_{\mathrm{III}}$):** Models an adversary launching fully chosen-key attacks to compromise aggregation soundness by forging a valid aggregate signature from invalid components.
- **Witness-Recovery Adversary ($A_{\mathrm{WR}}$):** Models a malicious insider capable of eavesdropping the public information transmitted over the open channel, aiming to recover a legitimate user’s witness $wit_{i}$ via computation, thereby breaking witness secrecy.
- **Identity-Grafting Adversary ($A_{\mathrm{IG}}$):** Models a revoked-but-malicious insider capable of obtaining a valid membership witness of an unrevoked user, aiming to graft legitimate identities onto its own pseudonym and generate signatures acceptable to the verifier.

To formally prove that our proposed scheme achieves *Existential Unforgeability under Chosen Message Attacks* (EUF-CMA) and *Aggregate Soundness*, we define three standard challenge-response games between a challenger C and three adversaries ($A_{I},A_{II},A_{III}$).

**Game I: Against Type-I Adversary ($A_{I}$).**

**Setup Phase:** C runs *Setup* to generate params and the master secret key *s*, sends params to $A_{I}$, and keeps *s* secret.

**Query Phase:** $A_{I}$ adaptively issues the following queries.

(1) *Create-User:* on input $PID_{i}$, C creates the user state and returns the public key $PK_{i}$.

(2) *Reveal-Partial-Private-Key:* on input $PID_{i}$, C returns the partial private key $ppk_{i}$.

(3) *Reveal-Secret-Value:* on input $PID_{i}$, C returns the user’s secret value $x_{i}$.

(4) *Reveal-Witness:* on input $PID_{i}$, C returns the accumulator witness $wit_{i}$.

(5) *Reveal-Public-Key:* on input $PID_{i}$, C returns the current $PK_{i}$.

(6) *Replace-Public-Key:* on input $\left(PID_{i},PK_{i}^{\prime }\right)$, C replaces the current $PK_{i}$ with $PK_{i}^{\prime }$.

(7) *Sign:* on input $\left(PID_{i},m_{i}\right)$, C returns a valid authentication transcript for $m_{i}$ under the *current* key material of $PID_{i}$.

**Forgery Phase:** $A_{I}$ outputs a tuple $\left(PID^{*},m^{*},\delta^{*},PK^{*},\pi^{*},T^{*}\right)$ and wins if the following hold.

(1) The transcript is accepted by the verification algorithm.

(2) $A_{I}$ never queried *Reveal-Partial-Private-Key* on $PID^{*}$.

(3) $A_{I}$ never queried *Sign* on $\left(PID^{*},m^{*}\right)$.

**Game II: Against Type-II Adversary ($A_{\mathrm{II}}$).**

**Setup Phase:** C runs *Setup* to obtain (params,s) and sends *both* (params,s) to $A_{II}$.

**Query Phase:** $A_{II}$ can adaptively issue *Create-User*, *Reveal-Secret-Value*, *Reveal-Witness*, *Reveal-Public-Key*, and *Sign* queries *as defined in Game I*. In this game, the *Replace-Public-Key* query is not allowed.

**Forgery Phase:** $A_{II}$ outputs $\left(PID^{*},m^{*},\delta^{*},PK^{*},\pi^{*},T^{*}\right)$ and wins if the following hold.

(1) The transcript is accepted by the verification algorithm.

(2) $A_{II}$ never queried *Reveal-Secret-Value* on $PID^{*}$.

(3) $A_{II}$ never queried *Sign* on $\left(PID^{*},m^{*}\right)$.

**Game III: Against Type-III Adversary ($A_{\mathrm{III}}$).**

**Setup Phase:** C runs *Setup* and sends params to $A_{III}$.

**Query Phase:** $A_{III}$ is allowed to adaptively issue the following queries.

(1) *Reveal-Full-Private-Key:* on input $PID_{i}$, C returns the full private key material of $PID_{i}$ (including $x_{i},ppk_{i}$).

(2) *AggVerify:* on input an aggregate candidate $\delta_{agg}$ with a set of message–identity pairs ${\left\{\left(m_{i},PID_{i}\right)\right\}}_{i=1}^{n}$, C runs *AggVerify* and returns the result.

**Forgery Phase:** $A_{III}$ outputs aggregate transcript $\delta_{agg}^{*}$ on ${\left\{\left(m_{i},PID_{i}\right)\right\}}_{i=1}^{n}$ and wins if the following hold.

(1) AggVerify accepts $\delta_{agg}^{*}$.

(2) There exists an index *i* such that the individual Verify rejects the corresponding component transcript for $\left(PID_{i},m_{i}\right)$.

## 4. Review of Shen et al.’s [8] Scheme

This section provides an overview of the certificateless authentication scheme proposed by Shen et al. [8], which integrates RSA accumulators to manage member dynamics in CLAS. The scheme consists of four primary phases: Setup, Registration, Authentication, and Membership Key Update.

### 4.1. Setup

The system initialization is collaboratively performed by the Trusted Authority (TA) and the Key Generation Center (KGC).

The TA selects global parameters {G,q,P} for the elliptic curve group and hash functions $H_{0}$∼$H_{5}$. Both authorities generate their master secret keys ($t,s\in Z_{q}^{*}$) and publish the corresponding public keys $T_{pub}=t\cdot P$ and $P_{pub}=s\cdot P$.

To support dynamic membership, the TA initializes the RSA accumulator by determining the modulus *N* and the base value $Acc_{init}=g$.

Then the system parameters $params=\{G,q,P,P_{pub},T_{pub},N,g,H_{0},H_{1},H_{2},H_{3},H_{4},H_{5}\}$ are broadcast to all entities.

### 4.2. Registration

In this phase, a new entity (SN or MS) interacts with the TA and KGC to register its identity and establish a valid key pair. The detailed procedure is executed as follows:

(1) A user ${\mathrm{User}}_{i}$ selects a random secret $x_{i}\in Z_{q}^{*}$ and computes $X_{i}=x_{i}\cdot P$. Additionally, the user chooses a unique random prime number $ID_{prime}$ from the prime set $Z_{prime}$. The tuple $\left(ID_{i},ID_{prime},X_{i}\right)$ is transmitted to the TA via a secure channel, where $ID_{i}$ represents the user’s real identity.

(2) Upon receiving the request, the TA sets the first part of the pseudonym as $PID_{i,1}=ID_{prime}$. To mask the real identity, the second part is computed as $PID_{i,2}=ID_{i}⊕H_{0}\left(g\cdot P_{pub},ID_{prime}\right)$. The full pseudonym is defined as $PID_{i}=\left(PID_{i,1},PID_{i,2}\right)$.

(3) The TA retrieves the current accumulator value $acc_{TA}$ to initialize the user’s witness $wit_{i}=acc_{TA}$. It then forwards the pseudonym $PID_{i}$ to the KGC.

(4) The KGC selects a random $r_{i}\in Z_{q}^{*}$ and computes $R_{i}=r_{i}\cdot P$. It then generates the partial private key using its master secret key *s* via the equation $ppk_{i}=r_{i}+s\cdot h_{1}\;\left(\mathrm{mod}\;q\right)$, where $h_{1}=H_{1}\left(P_{pub},X_{i},R_{i},PID_{i,2}\right)$. The KGC returns the tuple $\left(ppk_{i},R_{i},wit_{i}\right)$ to the user.

(5) Upon receiving the data, ${\mathrm{User}}_{i}$ computes the current accumulator value locally by $acc_{i}=wit_{i}^{PID_{i,1}}\;\left(\mathrm{mod}\;N\right)$. This value is stored for future membership verification.

(6) ${\mathrm{User}}_{i}$ computes a public witness $pwit_{i}=wit_{i}^{h_{2}}\;\left(\mathrm{mod}\;N\right)$, where $h_{2}=H_{2}\left(PID_{i},X_{i},acc_{i}\right)$. Finally, the user sets the full private key as $sk_{i}=\left(x_{i},ppk_{i},wit_{i}\right)$ and the full public key as $PK_{i}=\left(X_{i},R_{i},pwit_{i}\right)$.

### 4.3. Authentication

This phase involves the processes of generating and verifying individual signatures, as well as the aggregation performed by the Ward Node (WN).

#### 4.3.1. Signature Generation

To sign a medical data message $m_{i}$, the entity ${\mathrm{User}}_{i}$ executes the following operations:

(1) ${\mathrm{User}}_{i}$ selects a random number $u_{i}\in Z_{q}^{*}$ and computes $U_{i}=u_{i}\cdot P$.

(2) ${\mathrm{User}}_{i}$ computes two hash values required for the signature: $h_{3}=H_{3}\left(PID_{i},R_{i},U_{i}\right)$ and $h_{4}=H_{4}\left(PID_{i},m_{i},U_{i},PK_{i},T_{i}\right)$, where $T_{i}$ is the current timestamp.

(3) The signature $\sigma_{i}$ is calculated by: $\sigma_{i}=u_{i}+h_{3}\cdot x_{i}+h_{4}\cdot ppk_{i}\;\left(\mathrm{mod}\;q\right)$.

(4) Finally, ${\mathrm{User}}_{i}$ outputs the signature tuple $\delta_{i}=\left(\sigma_{i},U_{i}\right)$ and transmits the packet $\left(\delta_{i},m_{i},PID_{i},PK_{i},T_{i}\right)$ to the Ward Node.

#### 4.3.2. Signature Verification

Upon receiving the data packet, the verifier ${\mathrm{User}}_{j}$ performs the following checks:

(1) ${\mathrm{User}}_{j}$ verifies if the timestamp satisfies $|T_{j}-T_{i}|\;\leq \;\Delta T$. If the delay exceeds the threshold, the message is discarded.

(2) To ensure ${\mathrm{User}}_{i}$ is a valid member, ${\mathrm{User}}_{j}$ computes $h_{2}^{\prime }=H_{2}\left(PID_{i},X_{i},acc_{j}\right)$ and verifies if $pwit_{i}^{PID_{i,1}}\equiv acc_{j}^{h_{2}^{\prime }}\;\left(\mathrm{mod}\;N\right)$, where $acc_{j}$ is the locally accumulator value.

(3) If the membership is valid, ${\mathrm{User}}_{j}$ computes $h_{1}^{\prime }=H_{1}\left(P_{pub},X_{i},R_{i},PID_{i,2}\right)$, $h_{3}^{\prime }=H_{3}\left(PID_{i},R_{i},U_{i}\right)$ and $h_{4}^{\prime }=H_{4}\left(PID_{i},m_{i},U_{i},PK_{i},T_{i}\right)$. The signature is accepted if the equation $\sigma_{i}\cdot P=U_{i}+h_{3}^{\prime }\cdot X_{i}+h_{4}^{\prime }\cdot \left(R_{i}+h_{1}^{\prime }\cdot P_{pub}\right)$ holds.

#### 4.3.3. Aggregate Signature Generation

To reduce verification overhead, the Ward Node (WN) aggregates valid signatures destined for the Medical Server. The procedure is as follows:

(1) Upon receiving *n* message tuples, the WN checks the timestamp of each message. Any tuple with a delay exceeding the threshold ΔT is discarded.

(2) The WN verifies the validity of each sender using its local accumulator $acc_{WN}$. For each user ${\mathrm{User}}_{i}$, it computes $h_{2,i}^{\prime }=H_{2}\left(PID_{i},X_{i},acc_{WN}\right)$ and checks if the equation $pwit_{i}^{PID_{i,1}}\equiv acc_{WN}^{h_{2,i}^{\prime }}\;\left(\mathrm{mod}\;N\right)$ holds. Tuples failing this check are rejected.

(3) To resist information injection attacks, the WN generates a random vector $\eta =\{\eta_{2},\ldots ,\eta_{n}\}$, where each $\eta_{i}$ is a distinct small integer chosen from $\left[2,2^{l}\right]$. Note that the first signature remains unweighted.

(4) The WN computes the aggregate signature $\sigma_{agg}=\sigma_{1}+\sum_{i=2}^{n}\eta_{i}\cdot \sigma_{i}\;\left(\mathrm{mod}\;q\right)$. The final aggregate tuple $\delta_{agg}=\left(\sigma_{agg},{\left\{U_{i}\right\}}_{i=1}^{n}\right)$ is then transmitted with the current timestamp.

#### 4.3.4. Aggregate Signature Verification

Upon receiving the batch from the WN, the recipient ${\mathrm{User}}_{k}$ (Medical Server) verifies the aggregate signature as follows:

(1) ${\mathrm{User}}_{k}$ confirms that $|T_{k}-T_{agg}|\;\leq \;\Delta T$.

(2) For each i∈{1,…,n}, ${\mathrm{User}}_{k}$ computes the hash values $h_{3,i}^{\prime },h_{4,i}^{\prime }$ and $h_{1,i}^{\prime }$ based on the received public keys and messages.

(3) The aggregate signature is valid if the following equation holds: $\sigma_{agg}\cdot P=U_{agg}+X_{agg}+R_{agg}+P_{pub}\cdot \left(h_{1,1}^{\prime }h_{4,1}^{\prime }+\sum_{i=2}^{n}\eta_{i}h_{1,i}^{\prime }h_{4,i}^{\prime }\right)$, where the aggregated components are defined as $U_{agg}=U_{1}+\sum_{i=2}^{n}\eta_{i}U_{i}$, $X_{agg}=h_{3,1}^{\prime }X_{1}+\sum_{i=2}^{n}\eta_{i}h_{3,i}^{\prime }X_{i}$, and $R_{agg}=h_{4,1}^{\prime }R_{1}+\sum_{i=2}^{n}\eta_{i}h_{4,i}^{\prime }R_{i}$.

### 4.4. Membership Key Update

Shen et al. [8] used an RSA accumulator-based mechanism to manage dynamic membership. By broadcasting an Auxiliary Update message (AUX), the TA allows users to update their witnesses locally without having to reinitialize the system.

#### 4.4.1. AUX Distribution

The TA generates and broadcasts the AUX message based on the specific membership change scenario described below.

(1) When a new user with the prime identifier $PID_{n,1}$ joins the system (token=1), the TA selects a random $\psi \in Z_{q}^{*}$ to derive Ψ=ψ·P. It then computes the signature $Sig=\psi +\gamma_{n}\cdot t$, where $\gamma_{n}=H_{5}(P_{pub},\Psi ,PID_{n,1}\left||token\right)$. Subsequently, the TA updates the global accumulator by computing $acc_{TA}^{new}=acc_{TA}^{PID_{n,1}}\;\left(\mathrm{mod}\;N\right)$ and broadcasts the message $AUX=\{Sig,PID_{n,1},token,\Psi ,T_{m}\}$.

(2) When an existing user with $PID_{r,1}$ is revoked (token=0), the TA generates a corresponding signature using $\gamma_{r}=H_{5}(P_{pub},\Psi ,PID_{r,1}\left||token\right)$. In this case, the accumulator is updated by removing the revoked user’s prime representative: $acc_{TA}^{new}=acc_{TA}^{PID_{r,1}^{-1}\;\left(\mathrm{mod}\;\phi \left(S\right)\right)}\;\left(\mathrm{mod}\;N\right)$. Finally, the TA broadcasts $AUX=\{Sig,PID_{r,1},token,\Psi ,T_{m}\}$.

#### 4.4.2. Key Updating

Upon receiving AUX, a valid user ${\mathrm{User}}_{u}$ updates their keys as follows:

(1) ${\mathrm{User}}_{u}$ validates the timestamp $T_{m}$ and checking $Sig\cdot P=\Psi +\gamma \cdot T_{pub}$.

(2) If token≠0, ${\mathrm{User}}_{u}$ updates the witness and accumulator directly: $wit_{u}^{\prime }=wit_{u}^{PID_{n,1}}\;\left(\mathrm{mod}\;N\right)$ and $acc_{u}^{\prime }=acc_{u}^{PID_{n,1}}\;\left(\mathrm{mod}\;N\right)$.

(3) If token=0, ${\mathrm{User}}_{u}$ computes integers v,w satisfying $v\cdot PID_{u,1}+w\cdot PID_{r,1}=1$ via the Extended Euclidean algorithm. The values are updated as $wit_{u}^{\prime }=wit_{u}^{w}\cdot acc_{u}^{v}\;\left(\mathrm{mod}\;N\right)$ and $acc_{u}^{\prime }={\left(wit_{u}^{\prime }\right)}^{PID_{u,1}}\;\left(\mathrm{mod}\;N\right)$.

(4) ${\mathrm{User}}_{u}$ computes the new public witness $pwit_{u}^{\prime }={\left(wit_{u}^{\prime }\right)}^{H_{2}\left(PID_{u},X_{u},acc_{u}^{\prime }\right)}$ to finalize the key pair.

## 5. Cryptanalysis of Shen et al.’s [8] Scheme

Despite the security proofs provided in the random oracle model, a detailed cryptanalytic review has revealed that there are intrinsic design flaws in the scheme proposed by Shen et al. [8]. Specifically, it fails to ensure witness secrecy, sound revocation, and message unlinkability, all of which are critical for wireless medical sensor networks.

### 5.1. Witness Recovery Attack

Shen et al.’s [8] scheme security relies on the secrecy of the accumulator witness $wit_{i}$. To formalize the witness recovery attack, we consider a polynomial-time witness-recovery adversary $A_{WR}$, which represents a legitimate-but-malicious insider. $A_{WR}$ has the following capabilities:

(1) $A_{WR}$ can eavesdrop on the TA update broadcast information;

(2) $A_{WR}$ can eavesdrop on the transcripts of the same user across different sessions;

(3) $A_{WR}$ can perform polynomial-time computations on the collected data.

We say that $A_{WR}$ succeeds if it outputs a valid accumulator witness $wit_{i}$ of some legitimate target user $U_{i}$, thereby breaking witness secrecy.

Under this adversarial model, we show that an insider can derive the witness of a newly joining user from the broadcast information, and can also recover any target user’s $wit_{i}$ from public witnesses across multiple sessions. The concrete attacks are as follows.

#### 5.1.1. Recovery via Member-Joining Broadcast

This attack exploits the fact that the witness of a newly joining user is initialized as the current accumulator value. Hence, any registered user who can maintain the current accumulator can immediately obtain the new user’s witness.

**Attack procedure:**

Let A be a legitimate malicious user holding a valid identity prime $PID_{A,1}$ and witness $wit_{A}$. A can locally compute the current accumulator value as

$$acc_{\mathrm{old}}=wit_{A}^{\;PID_{A,1}}\;\left(\mathrm{mod}\;N\right).$$

When a new user ${\mathrm{User}}_{n}$ joins, the TA broadcasts the new accumulator element $PID_{n,1}$ and initializes the new witness as the current accumulator:

$$wit_{n}=acc_{\mathrm{old}}.$$

Therefore, upon intercepting the broadcast, the malicious user A immediately obtains the valid components $\left\{PID_{n,1},\;wit_{n}\right\}$.

#### 5.1.2. Recovery via Extended Euclidean Algorithm

In Shen et al.’s [8] scheme, user publishes $pwit_{i}=wit_{i}^{h_{2}}\;\left(\mathrm{mod}\;N\right)$, where $h_{2}$ is derived from the session transcript and varies with the accumulator acc. We show that a legitimate but malicious insider can recover the secret witness $wit_{i}$ by collecting two pwit values of the same target across different sessions and applying the Extended Euclidean Algorithm.

**Attack procedure:**

Let A be a legitimate malicious user. Following the protocol, A can obtain the current accumulator value acc and can link two broadcasts belonging to the same target ${\mathrm{User}}_{i}$.

In session $t_{1}$, A captures the target’s public witness

$$pwit_{1}=wit_{i}^{h_{2}}\;\mathrm{mod}\;N,\;h_{2}=H_{2}\left(PID_{i},X,i,acc_{1}\right),$$

After the system state is updated, the accumulator changes from $acc_{1}$ to $acc_{2}$. In a later session $t_{2}$, A captures another public witness from the same target:

$$pwit_{2}=wit_{i}^{h_{2}^{\prime }}\;\mathrm{mod}\;N,\;h_{2}^{\prime }=H_{2}\left(PID_{i},X,i,acc_{2}\right).$$

Under the random oracle model, the output of $H_{2}\left(\cdot \right)$ can be treated as a random integer. According to the result of Nymann et al. [36], the probability that two integers are coprime is $P=\mathrm{Pr}\left[\gcd\left(h_{2},h_{2}^{\prime }\right)=1\right]=6/\pi^{2}\approx 0.6079$. Thus, by collecting two sessions, the adversary obtains a coprime pair $\left(h_{2},h_{2}^{\prime }\right)$ with probability of about *P*. More generally, if the adversary collects *k* sessions, there are k2 candidate pairs, and the probability of obtaining at least one coprime pair can be estimated as Pr(∃i<j:gcd(hi,hj)=1)≈1−(1−P)k2. When k=3, this probability can be as high as $1-{\left(1-P\right)}^{3}\approx 0.9397$.

Once a coprime pair $\left(h_{2},h_{2}^{\prime }\right)$ is obtained, *A* runs the Extended Euclidean Algorithm to find integers (a,b) satisfying $ah_{2}+bh_{2}^{\prime }=1$. It then computes

$$pwit_{1}^{\;a}\cdot pwit_{2}^{\;b}\equiv wit_{i}^{ah_{2}}\cdot wit_{i}^{bh_{2}^{\prime }}\equiv wit_{i}^{ah_{2}+bh_{2}^{\prime }}\equiv wit_{i}\;\left(\mathrm{mod}\;N\right),$$

Consequently, the insider adversary recovers the secret witness $wit_{i}$ and obtains the valid components $\left\{PID_{i,1},\;wit_{i}\right\}$.

### 5.2. Identity Grafting Attack

In Shen et al.’s [8] scheme, the revocation mechanism relies solely on the invalidation of the accumulator element $PID_{i,1}$. To formalize the identity grafting attack, we consider a polynomial-time identity-grafting adversary $A_{IG}$, which represents a revoked-but-malicious insider. $A_{IG}$ has the following capabilities:

(1) $A_{IG}$ can retain its long-term secret and continue generating signatures;

(2) $A_{IG}$ can eavesdrop on the public information transmitted over the open channel;

(3) $A_{IG}$ can obtain a valid membership pair $\left(PID_{j,1},wit_{j}\right)$ of some unrevoked user $U_{j}$.

We say that $A_{IG}$ succeeds if, although its own identity has already been revoked, it can still output an authentication transcript that is accepted by the verifier.

Under this adversarial model, we show that $A_{IG}$ can graft the valid membership pair of $U_{j}$ into its own pseudonym and generate a valid signature, thereby bypassing the revocation check. The concrete attack procedure is given as follows.

**Attack Procedure:**

Let A be a legitimate but revoked user with identity $PID_{A}=\left(PID_{A,1},PID_{A,2}\right)$, public key components $\left(X_{A},R_{A}\right)$, and private keys $\left(x_{A},ppk_{A}\right)$. Note that $ppk_{A}$ satisfies $ppk_{A}=r_{A}+s\cdot h_{1,A}$, where $h_{1,A}=H_{1}\left(P_{pub},X_{A},R_{A},PID_{A,2}\right)$. After revocation, $PID_{A,1}$ is removed from the accumulator acc, invalidating A’s witness.

Using the witness recovery methods from Section 5.1, A obtains a valid membership pair $\left\{PID_{j,1},wit_{j}\right\}$ belonging to an active, non-revoked user ${\mathrm{User}}_{j}$. A then constructs a grafted identity $PID^{*}$ by combining the valid accumulator element of ${\mathrm{User}}_{j}$ with its own identity verification element:

$$PID^{*}=\left(PID_{1}^{*},PID_{2}^{*}\right):=\left(PID_{j,1},PID_{A,2}\right).$$

To pass the membership verification, A computes the updated hash $h_{2}^{*}$ and the corresponding public witness $pwit^{*}$ using the current accumulator acc:

$$h_{2}^{*}=H_{2}\left(PID^{*},X_{A},acc\right),\;pwit^{*}=wit_{j}^{h_{2}^{*}}\;\left(\mathrm{mod}\;N\right).$$

A generates a valid signature for a message *m* using its revoked (but mathematically valid) signing keys. A selects a random $u^{*}\in Z_{q}^{*}$, computes $U^{*}=u^{*}\cdot P$, and calculates the signature verification hashes based on the grafted identity $PID^{*}$:

$$h_{3}^{*}=H_{3}\left(PID^{*},R_{A},U^{*}\right),\;h_{4}^{*}=H_{4}\left(PID^{*},m,U^{*},PK_{A},T\right).$$

Using its original private keys $x_{A}$ and $ppk_{A}$, A computes the signature $\sigma^{*}$:

$$\sigma^{*}=u^{*}+h_{3}^{*}\cdot x_{A}+h_{4}^{*}\cdot ppk_{A}\;\left(\mathrm{mod}\;q\right).$$

Finally, A broadcasts the forged packet $M^{*}=\left(PID^{*},\sigma^{*},U^{*},X_{A},R_{A},pwit^{*},m,T\right)$.

**Verification Analysis:**

Upon receiving $M^{*}$, the verifier computes $h_{2}^{\prime }=H_{2}\left(PID^{*},X_{A},acc\right)$ and checks if

$${\left(pwit^{*}\right)}^{PID_{1}^{*}}\equiv acc^{h_{2}^{\prime }}\;\left(\mathrm{mod}\;N\right).$$

Since $PID_{1}^{*}=PID_{j,1}$ and $pwit^{*}$ is derived from the valid $wit_{j}$, this verification holds.

The verifier calculates $h_{1}^{\prime }=H_{1}\left(P_{pub},X_{A},R_{A},PID_{2}^{*}\right)$, $h_{3}^{\prime }=H_{3}\left(PID^{*},R_{A},U^{*}\right)$, and $h_{4}^{\prime }=H_{4}\left(PID^{*},m,U^{*},PK_{A},T\right)$. It then checks the equation:

$$\sigma^{*}\cdot P\overset{?}{=}U^{*}+h_{3}^{\prime }\cdot X_{A}+h_{4}^{\prime }\cdot \left(R_{A}+h_{1}^{\prime }\cdot P_{pub}\right).$$

Because $\sigma^{*}$ is generated using the correct private keys corresponding to $X_{A}$ and $R_{A}$, and the only hash value whose input the adversary cannot tamper with, namely $h_{1}^{\prime }$ (which is computed by the KGC and embedded into the partial private key ppk), is not bound to $PID_{1}^{*}$, the equality is fully satisfied during verification as well.

Thus, the verifier accepts $M^{*}$ as valid, the revocation is completely bypassed.

### 5.3. Message Linkability Attack

In Shen et al.’s [8] scheme, the identity-related fields carried in each packet, including the pseudonym $PID_{i}=\left(PID_{i,1},PID_{i,2}\right)$ and the public key components $\left(X_{i},R_{i}\right)$, remain static and repeatedly appear within a long observation window. Therefore, a passive eavesdropper can group packets by the repeated $PID_{i}$ or $\left(X_{i},R_{i}\right)$ and link multiple messages to the same sender. In wireless medical sensor networks, this enables the persistent tracking of a patient’s communication trace, allowing the adversary to infer healthcare-related habits and activity patterns from external features such as transmission timing and traffic direction. This violates unlinkability.

Moreover, message linkability directly enables the witness recovery attack described in Section 5.1. This attack requires linking two public witnesses of the same user across different sessions; thus, static identity fields both leak privacy and provide the necessary condition for cross-session witness recovery.

## 6. The Proposed Protocol

To remedy the intrinsic design flaws in Shen et al.’s [8] scheme, the main improvements of our protocol are summarized as follows (Table 2).

- In the certificateless partial private key $ppk_{i}$, we bind the prime pseudonym $PID_{i,1}$ to $\left(PID_{i,2},X_{i},R_{i}\right)$ via a hash function, preventing identity grafting attacks.
- We adopt a non-interactive zero-knowledge membership proof, enabling a user to prove the relation $acc_{j}=wit_{i}^{PID_{i,1}}\;\mathrm{mod}\;N$ while keeping $wit_{i}$ hidden, and prevent witness recovery attacks based on extended Euclid algorithm.
- We introduce a per-user pseudonym pool with periodic switching, and TA broadcasts only a product-form update exponent *E* for the pool, which prevents message linkability and witness recovery attacks based on broadcast updates.
- We redesign the signature computation into a single-hash-driven linear form, improving the efficiency of signature generation and verification.

### 6.1. Setup

With the security parameter λ, TA and KGC initialize the algebraic environments, public key, hash interfaces, and the RSA-accumulator for the certificateless authentication system, while keeping their secret values private.

(1) TA selects a cyclic group G with |G|=q and generator *P*. It chooses $t\in Z_{q}^{*}$ and publishes the corresponding public key $T_{pub}=t\cdot P$.

(2) KGC chooses its master secret $s\in Z_{q}^{*}$ and releases the master public key $P_{pub}=s\cdot P$.

(3) TA instantiates the following hash functions that are used in the protocol:

$H_{0}:G\times Z_{N}^{*}\to {\left\{0,1\right\}}^{*}$,

$H_{1}:G^{3}\times {\left({\left\{0,1\right\}}^{*}\right)}^{2}\to Z_{q}^{*}$,

$H_{2}:G^{2}\times {\left(Z_{N}^{*}\right)}^{2}\times {\left({\left\{0,1\right\}}^{*}\right)}^{2}\to {\left\{0,1\right\}}^{*}$,

$H_{3}:G^{3}\times {\left(Z_{N}^{*}\right)}^{3}\times {\left({\left\{0,1\right\}}^{*}\right)}^{4}\to Z_{q}^{*}$,

$H_{4}:G^{2}\times Z_{N}^{*}\times {\left({\left\{0,1\right\}}^{*}\right)}^{3}\to Z_{q}^{*}$.

(4) TA generates the RSA modulus for the accumulator by selecting two large primes B,D and setting N=BD. It then initializes the accumulator by choosing a random generator $g\in Z_{N}^{*}$ and setting $acc_{\mathrm{init}}=g$.

(5) Finally, TA publishes the system parameters as $params=\{G,q,P,N,T_{pub},P_{pub},H_{0},H_{1},H_{2},H_{3},H_{4}\}$.

### 6.2. Registration

Upon receiving a registration request from a user ${\mathrm{User}}_{i}$ with real identity $ID_{i}$, the TA allocates a pseudonym pool and accumulator-membership witnesses, while the KGC issues the corresponding certificateless partial private keys that are bound to $PID_{i,1}$ to prevent *Identity Grafting Attack*.

(1) ${\mathrm{User}}_{i}$ randomly selects *n* secret values ${\left\{x_{k}\right\}}_{k=1}^{n}\in Z_{q}^{*}$ and computes the corresponding public keys $X_{k}=x_{k}\cdot P$ for each k∈{1,…,n}. It then sends $\left(ID_{i},{\left\{X_{k}\right\}}_{k=1}^{n}\right)$ to TA through a secure channel.

(2) For the *k*-th public key of ${\mathrm{User}}_{i}$ (for k=1,…,n), TA chooses a fresh prime representative $ID_{k,\mathrm{prime}}$ and sets $PID_{k,1}=ID_{k,\mathrm{prime}}$, where $\gcd(ID_{k,\mathrm{prime}},N)=1$ holds. It then computes $PID_{k,2}=ID_{i}⊕H_{0}\;\left(t\cdot X_{k},ID_{k,\mathrm{prime}}\right)$, and stores the tracing record in its private table for future identity recovery.

(3) Then TA computes the batch exponent $E_{i}=\prod_{k=1}^{n}PID_{k,1}\;\mathrm{mod}\;\varphi \left(N\right)$, and performs accumulator update as $acc_{new}=acc_{old}^{E_{i}}\;\mathrm{mod}\;N$. For each k∈{1,…,n}, TA further computes the witness exponent $E_{k}=\prod_{\ell =1,\ell \neq k}^{n}PID_{\ell ,1}\;\mathrm{mod}\;\varphi \left(N\right)$ and derives the corresponding membership witness as $wit_{k}=acc_{old}^{E_{k}}\;\mathrm{mod}\;N$. Then TA sends ${\left\{\left(PID_{k,1},PID_{k,2},wit_{k}\right)\right\}}_{k=1}^{n}$ to ${\mathrm{User}}_{i}$, sends ${\left\{\left(PID_{k,1},PID_{k,2},X_{k}\right)\right\}}_{k=1}^{n}$ to KGC via a secure channel.

(4) Upon receiving the message from TA, for each *k*, KGC randomly selects $r_{k}\in Z_{q}^{*}$ and computes $R_{k}=r_{k}\cdot P$. It then computes $h_{1}=H_{1}\left(P_{pub},X_{k},R_{k},PID_{k,1},PID_{k,2}\right)$, and outputs the certificateless partial private key $ppk_{k}=\left(r_{k}+h_{1}\cdot s\right)\;\mathrm{mod}\;q$. KGC securely returns ${\left\{\left(R_{k},ppk_{k}\right)\right\}}_{k=1}^{n}$ to ${\mathrm{User}}_{i}$, and ${\mathrm{User}}_{i}$ keeps ${\left\{ppk_{k}\right\}}_{k=1}^{n}$ secret.

(5) After receiving the messages from TA and KGC, ${\mathrm{User}}_{i}$ first verifies the correctness of each $ppk_{k}$ by checking whether $ppk_{k}\cdot P\overset{?}{=}R_{k}+h_{1}\cdot P_{pub}$, where $h_{1}=H_{1}\left(P_{pub},X_{k},R_{k},PID_{k,1},PID_{k,2}\right)$. Then, ${\mathrm{User}}_{i}$ randomly selects an index $k^{*}\in \left\{1,\ldots ,n\right\}$ and computes the latest accumulator value as $acc_{\mathrm{new}}=wit_{k^{*}}^{PID_{k^{*},1}}\;\mathrm{mod}\;N.$ Finally, ${\mathrm{User}}_{i}$ stores $acc_{\mathrm{new}}$ and ${\left\{\left(PID_{k,1},PID_{k,2},wit_{k}\right)\right\}}_{k=1}^{n}$ locally for subsequent authentication.

(6) For each *k*, ${\mathrm{User}}_{i}$ sets the full private key as $sk_{k}=\left(x_{k},ppk_{k}\right)$, the pseudo-identity as $PID_{k}=\left(PID_{k,1},PID_{k,2}\right)$, and the corresponding public key as $PK_{k}=\left(X_{k},R_{k}\right)$.

### 6.3. Authentication

To prevent message linkability attacks, ${\mathrm{User}}_{i}$ maintains a pool of *n* pseudonym instances ${\left\{\left(PID_{k,1},PID_{k,2},wit_{k},PK_{k}\right)\right\}}_{k=1}^{n}$ and periodically switches among them, denoting the selected instance as $PID_{i}=\left(PID_{i,1},PID_{i,2}\right)$, $PK_{i}=\left(X_{i},R_{i}\right)$, and $wit_{i}$. The verifier validates the attached zero-knowledge membership proof and the signature with respect to the latest accumulator value $acc_{j}$.

#### 6.3.1. Zero-Knowledge Membership Proof Generation

To keep $wit_{i}$ secret while proving valid membership, ${\mathrm{User}}_{i}$ generates a non-interactive zero-knowledge membership proof $\pi_{i}$ for the relation $acc_{j}\equiv wit_{i}^{PID_{i,1}}\;\left(\mathrm{mod}\;N\right)$. Such a proof follows the standard three-move structure: (1) commitment, (2) challenge, and (3) response. In our design, non-interactivity is achieved via the Fiat–Shamir transformation [35], where the challenge is generated by a hash function. The detailed procedure is as follows.

(1) ${\mathrm{User}}_{i}$ picks a random $a_{i}\in Z_{N}^{*}$ and computes the commitment $A_{i}=a_{i}^{PID_{i,1}}\;\mathrm{mod}\;N$.

(2) ${\mathrm{User}}_{i}$ computes the challenge $c_{i}=H_{2}\left(X_{i},R_{i},A_{i},acc_{j},PID_{i,1},PID_{i,2}\right)$.

(3) ${\mathrm{User}}_{i}$ computes the response $C_{i}=a_{i}\cdot wit_{i}^{c_{i}}\;\mathrm{mod}\;N$.

Finally, ${\mathrm{User}}_{i}$ outputs the proof $\pi_{i}=\left(A_{i},C_{i}\right)$. Since the challenge does not involve $m_{i}$ and $T_{i}$, ${\mathrm{User}}_{i}$ can precompute the proof during idle time, which significantly reduces the online overhead during authentication.

#### 6.3.2. Signature Generation

${\mathrm{User}}_{i}$ generates a signature using the partial private key and the user secret key.

(1) ${\mathrm{User}}_{i}$ randomly selects $u_{i}\in Z_{q}^{*}$ and computes $U_{i}=u_{i}\cdot P$.

(2) ${\mathrm{User}}_{i}$ computes the hash $h_{3}=H_{3}\left(U_{i},X_{i},R_{i},A_{i},C_{i},acc_{j},PID_{i,1},PID_{i,2},m_{i},T_{i}\right)$.

(3) ${\mathrm{User}}_{i}$ computes the signature scalar $\sigma_{i}=u_{i}+h_{3}\cdot \left(x_{i}+ppk_{i}\right)\;\mathrm{mod}\;q$, and outputs $\delta_{i}=\left(\sigma_{i},U_{i}\right)$ as the signature.

(4) Finally, ${\mathrm{User}}_{i}$ sends $\left(\delta_{i},m_{i},PID_{i},PK_{i},\pi_{i},T_{i}\right)$ to the verifier.

#### 6.3.3. Signature Verification

Upon receiving the authentication message, the verifier first checks the membership proof, and then verifies the signature.

(1) Parse the received tuple as $\left(\delta_{i},m_{i},PID_{i},PK_{i},\pi_{i},T_{i}\right)$, where $\delta_{i}=\left(\sigma_{i},U_{i}\right)$, $PID_{i}=\left(PID_{i,1},PID_{i,2}\right)$, $PK_{i}=\left(X_{i},R_{i}\right)$, and $\pi_{i}=\left(A_{i},C_{i}\right)$. Use the latest accumulator value $acc_{j}$ maintained locally.

(2) If $|T_{ver}-T_{i}|<\Delta T$, compute $c_{i}=H_{2}\left(X_{i},R_{i},A_{i},acc_{j},PID_{i,1},PID_{i,2}\right)$ and regard the ZKP as valid only when $C_{i}^{PID_{i,1}}\overset{?}{=}A_{i}\cdot acc_{j}^{c_{i}}\;\mathrm{mod}\;N$.

(3) Compute $h_{1}=H_{1}\left(P_{pub},X_{i},R_{i},PID_{i,1},PID_{i,2}\right)$.

(4) Compute $h_{3}=H_{3}\left(U_{i},X_{i},R_{i},A_{i},C_{i},acc_{j},PID_{i,1},PID_{i,2},m_{i},T_{i}\right)$.

(5) The signature is accepted only when $\sigma_{i}\cdot P\overset{?}{=}U_{i}+h_{3}\cdot \left(R_{i}+X_{i}\right)+\left(h_{3}\cdot h_{1}\right)\cdot P_{pub}$.

#### 6.3.4. Aggregate Signature Generation

In scenarios involving high traffic, the Ward Node (WN) aggregates multiple authenticated tuples destined for the same verifier, thereby reducing the verification overhead.

(1) Upon receiving ${\left\{\left(\delta_{i},m_{i},PID_{i},PK_{i},\pi_{i},T_{i}\right)\right\}}_{i=1}^{n}$ from different ${\mathrm{User}}_{i}$, WN first checks timestamps and discards any tuple with $|T_{WN}-T_{i}|>\Delta T$.

(2) For each remaining tuple, WN performs the individual checks using the locally stored latest accumulator value $acc_{j}$: compute $c_{i}=H_{2}\left(X_{i},R_{i},A_{i},acc_{j},PID_{i,1},PID_{i,2}\right)$ and accept $\pi_{i}=\left(A_{i},C_{i}\right)$ only when $C_{i}^{PID_{i,1}}\overset{?}{=}A_{i}\cdot acc_{j}^{c_{i}}\;\mathrm{mod}\;N$.

(3) WN generates a coefficient vector $\eta =\{\eta_{2},\eta_{3},\ldots ,\eta_{n}\}$ of random integers, where, for example, $\eta_{i}\in \left[2,2^{\ell }\right]$ and *ℓ* is determined by the batch size, and sets $\eta_{1}=1$ by default.

(4) WN computes the aggregate scalar as $\sigma_{agg}=\sigma_{1}+\sum_{i=2}^{n}\eta_{i}\sigma_{i}\;\mathrm{mod}\;q$, and outputs the aggregate signature as $\delta_{agg}=\left(\sigma_{agg},{\left\{U_{i}\right\}}_{i=1}^{n},\eta \right)$, which is then delivered to the verifier together with the corresponding batch payload and the aggregation timestamp $T_{agg}$.

#### 6.3.5. Aggregate Signature Verification

Upon receiving the aggregated packet, the verifier validates the batch using a single aggregate equation in relation to the accumulator value, $acc_{j}$, which is maintained locally.

(1) Check the freshness of the aggregation timestamp $T_{agg}$ and abort if $|T_{ver}-T_{agg}|>\Delta T$, where $T_{ver}$ is the verifier’s current local time.

(2) For each i∈{1,…,n}, compute $h_{1,i}=H_{1}\left(P_{pub},X_{i},R_{i},PID_{i,1},PID_{i,2}\right)$ and $h_{3,i}=H_{3}\left(U_{i},X_{i},R_{i},A_{i},C_{i},acc_{j},PID_{i,1},PID_{i,2},m_{i},T_{i}\right)$, and set $\eta_{1}=1$.

(3) Compute the aggregate points $U_{agg}=U_{1}+\sum_{i=2}^{n}\eta_{i}U_{i}$, $V_{agg}=h_{3,1}\left(R_{1}+X_{1}\right)+\sum_{i=2}^{n}\eta_{i}h_{3,i}\left(R_{i}+X_{i}\right)$, and $W_{agg}=\left(h_{3,1}h_{1,1}+\sum_{i=2}^{n}\eta_{i}h_{3,i}h_{1,i}\right)\cdot P_{pub}$.

(4) The aggregate signature is regarded as valid only when the following equation holds: $\sigma_{agg}\cdot P\overset{?}{=}U_{agg}+V_{agg}+W_{agg}$.

### 6.4. Membership Witness Update

To support dynamic membership management, the TA broadcasts an authenticated auxiliary message to synchronize the accumulator value and enable legitimate entities to update their membership witnesses after member joining or revocation events.

(1) Let the current accumulator value be $acc_{old}\in QR_{N}$, and the TA computes the updated value as $acc_{new}\in QR_{N}$ after processing the latest event.

(2) For batch joining, the TA defines the joining exponent $E_{add}=\prod_{\ell \in J}PID_{\ell ,1}$, where J denotes the pools of newly joined pseudonym primes.

(3) For batch revocation, the TA defines the revocation exponent $E_{rev}=\prod_{\ell \in R}PID_{\ell ,1}$, where R denotes the pools of revoked pseudonym primes.

#### 6.4.1. AUX Distribution

According to the membership-change scenario indicated by token, the TA prepares the AUX message. The detailed distribution process performed by the TA is as follows.

(1) Batch joining (token=1): when a set of new pseudonym primes ${\left\{PID_{\ell ,1}\right\}}_{\ell \in J}$ of ${\mathrm{User}}_{i}$ is admitted, TA computes the joining exponent $E_{add}=\prod_{\ell \in J}PID_{\ell ,1}$ and updates the accumulator as $acc_{new}=acc_{old}^{E_{add}}\;\mathrm{mod}\;N$.

(2) Batch revocation (token=0): when a revocation report $\left(\delta_{i},m_{i},PID_{i},PK_{i},\pi_{i},T_{i}\right)$ is submitted, the TA recovers the real identity by the inline formula $ID_{i}=PID_{i,2}⊕H_{0}\left(t\cdot X_{i},PID_{i,1}\right)$. The TA then computes the revocation exponent over all pseudonym primes of this user as $E_{rev}=\prod_{\ell \in R}PID_{\ell ,1}$ and updates the accumulator as $acc_{new}=acc_{old}^{E_{rev}^{-1}}\;\mathrm{mod}\;N$.

(3) The TA randomly selects $\psi \in Z_{q}^{*}$, Ψ=ψ·P, sets $E=E_{add}$ if token=1 and $E=E_{rev}$ if token=0, then it computes $h_{4}=H_{4}\left(P_{pub},\Psi ,E,acc_{new},token,T_{m}\right)$, $\mathrm{Sig}=\psi +h_{4}\cdot t\;\mathrm{mod}\;q$, where $T_{m}$ is the current timestamp, finally broadcasting $AUX=\{\mathrm{Sig},E,acc_{new},token,\Psi ,T_{m}\}$ over the public channel.

Notably, broadcasting only the product-form exponent *E* for a user’s pseudonym pool makes it computationally infeasible to recover each individual $PID_{\left(l,1\right)}$ from *E* under the large-integer factorization assumption, which prevents the eavesdropping-based witness recovery in Shen et al.’s [8] public update broadcast.

#### 6.4.2. Witness Updating

After receiving $AUX=\{\mathrm{Sig},E,acc_{new},token,\Psi ,T_{m}\}$, a legitimate entity updates its locally stored accumulator value and membership witnesses under the latest system state.

(1) The receiver checks the freshness of the update by verifying $|T_{n}-T_{m}|\;\leq \;\Delta T$, where $T_{n}$ denotes its current local timestamp. If the check fails, it discards AUX.

(2) The receiver computes $h_{4}=H_{4}\left(P_{pub},\Psi ,E,acc_{new},token,T_{m}\right)$ and checking $\mathrm{Sig}\cdot P\overset{?}{=}\Psi +h_{4}\cdot T_{pub}$. If the equation does not hold, it discards AUX.

(3) Updating after joining (token=1): for each stored witness wit bound to a pseudonym prime $PID_{1}$ in the pseudonym pool, updates $wit\leftarrow wit^{E_{add}}\;\mathrm{mod}\;N$.

(4) Updating after revocation (token=0): since $\gcd(PID_{1},E_{rev})=1$ holds for any non-revoked user, for each stored witness wit bound to a pseudonym prime $PID_{1}$, the receiver computes (v,w) such that $v\cdot PID_{1}+w\cdot E_{rev}=1$ and updates witness $wit\leftarrow {\left(acc_{new}\right)}^{v}\cdot {\left(wit\right)}^{w}\;\mathrm{mod}\;N$.

Above revocation updating follows from Bézout’s identity $v\cdot PID_{1}+w\cdot E_{rev}=1$ and the relation $acc_{old}=acc_{new}^{E_{rev}}\;\mathrm{mod}\;N$. Let $wit^{\prime }={\left(acc_{new}\right)}^{v}\cdot {\left(wit\right)}^{w}\;\mathrm{mod}\;N$, then

$$\begin{array}{cc} {\left(wit^{\prime }\right)}^{PID_{1}} & ={\left(acc_{new}\right)}^{v\cdot PID_{1}}\cdot {\left(wit^{PID_{1}}\right)}^{w}\;\mathrm{mod}\;N\\ & ={\left(acc_{new}\right)}^{v\cdot PID_{1}}\cdot {\left(acc_{old}\right)}^{w}\;\mathrm{mod}\;N\\ & ={\left(acc_{new}\right)}^{v\cdot PID_{1}}\cdot {\left(acc_{new}^{E_{rev}}\right)}^{w}\;\mathrm{mod}\;N\\ & ={\left(acc_{new}\right)}^{v\cdot PID_{1}+w\cdot E_{rev}}\;\mathrm{mod}\;N\\ & =acc_{new}\;\mathrm{mod}\;N. \end{array}$$

which ensures the consistency of $wit^{\prime }$ with $acc_{new}$. For a revoked pseudonym prime, $\gcd(PID_{1},E_{rev})\neq 1$ holds and thus the coefficients (v,w) do not exist, so the corresponding witness cannot be updated.

## 7. Security Analysis

### 7.1. Formal Security Proof

Since the successful forgery of an aggregate signature implies the forgery of at least one constituent individual signature, we prove the EUF-CMA security of our proposed scheme in Theorems 1 and 2. We also prove the aggregate soundness against fully chosen-key attacks in Theorem 3.

**Theorem 1.**  *If there exists a Type-I adversary $A_{I}$ that can output a valid forgery with advantage $\epsilon_{I}$ in Game I, then there exists a PPT simulator $B_{1}$ that solves the ECDLP with non-negligible probability of at least $\epsilon_{I}^{2}/\left(e\;\left(Q_{cu}+1\right)\;\left(Q_{h1}+Q_{sig}+1\right)\right)$, where $Q_{cu}$, $Q_{h1}$, and $Q_{sig}$ denote the maximum numbers of* Create-User*, $H_{1}$, and* Sign *queries, respectively.*

**Proof.**  Given an ECDLP instance (P,aP), $B_{1}$ sets $P_{pub}=aP$ and runs $A_{I}$ as a subroutine in the random oracle model to extract *a*.

*Setup:* $B_{1}$ selects $t\leftarrow Z_{q}^{*}$ randomly and sets $T_{pub}=t\cdot P$. It initializes the RSA-accumulator parameters exactly as in the real protocol and outputs params. It maintains lists $L_{ID}$, $L_{PK}$, $L_{1}$, $L_{2}$, and $L_{3}$ for consistency, and picks $\tau \leftarrow \{1,2,\ldots ,Q_{cu}+1\}$ to guess the target identity.

*Query Stage:* $A_{I}$ adaptively issues the following queries.

(1) *$H_{1}$ Query:* On input $\left(P_{pub},X_{i},R_{i},PID_{i,1},PID_{i,2}\right)$, if there exists a record in $L_{1}$, $B_{1}$ returns the stored $h_{1,i}$; otherwise it selects $h_{1,i}\leftarrow Z_{q}^{*}$ randomly, stores the tuple into $L_{1}$, and returns $h_{1,i}$.

(2) *$H_{2}$ Query:* On input $\left(X_{i},R_{i},A_{i},acc_{j},PID_{i,1},PID_{i,2}\right)$, if there exists a record in $L_{2}$, $B_{1}$ returns the stored $c_{i}$; otherwise it selects $c_{i}\leftarrow {\left\{0,1\right\}}^{*}$ randomly, stores the tuple into $L_{2}$, and returns $c_{i}$.

(3) *$H_{3}$ Query:* On input $\left(U_{i},X_{i},R_{i},A_{i},C_{i},acc_{j},PID_{i,1},PID_{i,2},m_{i},T_{i}\right)$, if there exists a record in $L_{3}$, $B_{1}$ returns the stored $h_{3,i}$; otherwise it selects $h_{3,i}\leftarrow Z_{q}^{*}$ randomly, stores the tuple into $L_{3}$, and returns $h_{3,i}$.

(4) *Create-User Query:* On input $PID_{i}$, if $PID_{i}\in L_{ID}$ return ⊥; otherwise $B_{1}$ performs as follows.

(i) $B_{1}$ selects $x_{i},ppk_{i},h_{1,i}\leftarrow Z_{q}^{*}$ randomly and sets $X_{i}=x_{i}\cdot P$.

(ii) $B_{1}$ sets $R_{i}=ppk_{i}\cdot P-h_{1,i}\cdot P_{pub}$ so that $ppk_{i}\cdot P=R_{i}+h_{1,i}\cdot P_{pub}$ holds.

(iii) $B_{1}$ programs $H_{1}\left(P_{pub},X_{i},R_{i},PID_{i,1},PID_{i,2}\right)=h_{1,i}$ by inserting the tuple into $L_{1}$.

(iv) $B_{1}$ assigns consistent pseudonym components and an accumulator witness $wit_{i}$ as in registration, stores $\left(PID_{i},X_{i},R_{i},x_{i},ppk_{i},wit_{i}\right)$ into $L_{ID}$ and $\left(PID_{i},PK_{i}=\left(X_{i},R_{i}\right)\right)$ into $L_{PK}$. If this is the τ-th distinct creation, set $PID^{*}\leftarrow PID_{i}$. Finally return $PK_{i}$.

(5) *Reveal Queries:* If the queried $PID_{i}$ has no record, $B_{1}$ first runs *Create-User* on $PID_{i}$. Then it answers the following:

(i) *Reveal-Public-Key* returns the current $PK_{i}$ from $L_{PK}$;

(ii) *Reveal-Secret-Value* returns $x_{i}$;

(iii) *Reveal-Partial-Private-Key* returns $ppk_{i}$ unless $PID_{i}=PID^{*}$, $B_{1}$ aborts;

(iv) *Reveal-Witness* returns $wit_{i}$.

(6) *Replace-Public-Key Query:* On input $\left(PID_{i},PK_{i}^{\prime }\right)$, $B_{1}$ updates $L_{PK}$ with $PK_{i}^{\prime }$ and marks the effective signing secret of $PID_{i}$ as unknown.

(7) Sign Query: On input $\left(PID_{i},m_{i}\right)$, $B_{1}$ obtains the current public key $PK_{i}=\left(X_{i},R_{i}\right)$ from $L_{PK}$ and parses $\left(PID_{i,1},PID_{i,2},wit_{i}\right)$ from $L_{ID}$, and then generates a valid membership proof $\pi_{i}=\left(A_{i},C_{i}\right)$ under the current accumulator value as in the real protocol.

If $PK_{i}$ has not been replaced and $PID_{i}\neq PID^{*}$, $B_{1}$ returns a real signature transcript as in the protocol. Otherwise, $B_{1}$ simulates a valid signature transcript as follows: it queries $H_{1}\left(P_{pub},X_{i},R_{i},PID_{i,1},PID_{i,2}\right)$ to obtain $h_{1,i}$, sets $Y_{i}=\left(X_{i}+R_{i}\right)+h_{1,i}\cdot P_{pub}$, selects $\sigma_{i},h_{3,i}\leftarrow Z_{q}^{*}$ randomly, sets $U_{i}=\sigma_{i}\cdot P-h_{3,i}\cdot Y_{i}$, programs $H_{3}\left(U_{i},X_{i},R_{i},A_{i},C_{i},acc_{j},PID_{i,1},PID_{i,2},m_{i},T_{i}\right)=h_{3,i}$ in $L_{3}$, and returns $\delta_{i}=\left(\sigma_{i},U_{i}\right)$ together with $\left(PID_{i},PK_{i},\pi_{i},T_{i}\right)$.

*Forgery:* After all the queries have been completed, $A_{I}$ outputs a valid forgery $\left(PID^{*},m^{*},\delta^{*},PK^{*},\pi^{*},T^{*}\right)$, where $\delta^{*}=\left(\sigma^{*},U^{*}\right)$, $PK^{*}=\left(X^{*},R^{*}\right)$, and $\pi^{*}=\left(A^{*},C^{*}\right)$.

If $PID^{*}\neq PID_{i}^{*}$, then $B_{1}$ outputs ⊥ and aborts.

Otherwise, let $h_{1}^{*}$ be the output of $H_{1}\left(P_{pub},X^{*},R^{*},PID_{1}^{*},PID_{2}^{*}\right)$ and let $h_{3}^{*}$ be the output of $H_{3}\left(U^{*},X^{*},R^{*},A^{*},C^{*},acc_{j},PID_{1}^{*},PID_{2}^{*},m^{*},T^{*}\right)$ recorded in $L_{3}$.

By the correctness of verification, the forged transcript satisfies

$$\left\{\begin{array}{cc} \sigma^{*}\cdot P & =U^{*}+h_{3}^{*}\cdot \left(X^{*}+R^{*}\right)+h_{3}^{*}\cdot h_{1}^{*}\cdot P_{pub},\\ \sigma^{{*}^{\prime }}\cdot P & =U^{*}+h_{3}^{*}\cdot \left(X^{*}+R^{*}\right)+h_{3}^{*}\cdot h_{1}^{{*}^{\prime }}\cdot P_{pub}, \end{array}\right.$$

where $\delta^{{*}^{\prime }}=\left(\sigma^{{*}^{\prime }},U^{*}\right)$ is obtained by rewinding $A_{I}$ and replaying the random oracle $H_{1}$ such that $h_{1}^{*}\neq h_{1}^{{*}^{\prime }}$ while the other transcript components remain unchanged.

Subtracting the above two equations yields

$$\left(\sigma^{*}-\sigma^{{*}^{\prime }}\right)\cdot P=h_{3}^{*}\cdot \left(h_{1}^{*}-h_{1}^{{*}^{\prime }}\right)\cdot P_{pub}=h_{3}^{*}\cdot \left(h_{1}^{*}-h_{1}^{{*}^{\prime }}\right)\cdot aP.$$

Therefore, $B_{1}$ outputs

$$a\equiv \left(\sigma^{*}-\sigma^{{*}^{\prime }}\right)\cdot {\left(h_{3}^{*}\cdot \left(h_{1}^{*}-h_{1}^{{*}^{\prime }}\right)\right)}^{-1}\;\left(\mathrm{mod}\;q\right)$$

as the solution to the given ECDLP instance.

Let $E_{1}$ denote the event that the simulation does not abort in the query stage, $E_{2}$ denote the event that $B_{1}$ correctly guesses the target identity, and $E_{3}$ denote the event that $B_{1}$ obtains two valid forgeries with different $H_{1}$ outputs by the Forking Lemma [37]. Then $\mathrm{Pr}\left[E_{2}\right]\geq 1/\left(Q_{cu}+1\right)$ and $\mathrm{Pr}\left[E_{3}|E_{1}\wedge E_{2}\right]\geq \epsilon_{I}^{2}/\left(e\;(Q_{h1}+Q_{sig}+1)\right)$. Hence, $B_{1}$ solves the ECDLP with probability of at least $\epsilon_{I}^{2}/\left(e\;\left(Q_{cu}+1\right)\;\left(Q_{h1}+Q_{sig}+1\right)\right)$.

Therefore, the above reduction shows that if a Type-I adversary can produce a valid forgery in Game I, then one can construct a PPT algorithm to solve the ECDLP with non-negligible probability. Since the ECDLP is assumed to be hard, such a Type-I forgery is infeasible for any PPT adversary. Hence, the proposed scheme is secure against Type-I adversarial forgery attacks. □

**Theorem 2.**  *In the random oracle model, if there exists a Type-II adversary $A_{II}$ that can output a valid forgery in Game II with non-negligible advantage $\epsilon_{II}$, then one can construct a PPT algorithm that solves the ECDLP with non-negligible probability.*

**Proof.**  The proof is similar to that of Theorem 1 and thus is omitted for brevity. It is only necessary to mention that in Game II, $A_{II}$ does not initiate the *Replace-Public-Key* query. Following the same reduction strategy as in Theorem 1, if $A_{II}$ can produce a valid forgery with non-negligible advantage, then one can construct a PPT algorithm that solves the ECDLP with non-negligible probability.

Since the ECDLP is assumed to be hard, such a Type-II forgery is infeasible for the PPT adversary. Hence, the proposed scheme is secure against Type-II adversarial forgery attacks. □

**Theorem 3.**  *If there exists a Type-III adversary $A_{III}$ that wins Game III with advantage $\epsilon_{III}$, then there exists a PPT simulator $B_{3}$ that breaks the soundness guarantee of the simplified small exponent test (SET) [38] with non-negligible probability. In particular, $\epsilon_{III}\leq \left(Q_{av}+1\right)\cdot 2^{-\ell }$, where $Q_{av}$ denotes the maximum number of* AggVerify *queries and ℓ is the bit-length parameter used to sample the aggregation coefficients.*

**Proof.**  Given a challenger that samples the aggregation coefficients as required by the simplified SET, $B_{3}$ runs $A_{III}$ as a subroutine and uses any successful information-injection forgery to violate the SET soundness bound.

*Setup:* $B_{3}$ runs *Setup* to generate params and provides params to $A_{III}$. It maintains a user state list $L_{ID}$ and initializes all hash-oracle lists as in the real protocol.

*Query Stage:* $A_{III}$ adaptively issues the following queries.

(1) *Reveal-Full-Private-Key:* on input $PID_{i}$, if $PID_{i}$ has no record, $B_{3}$ internally creates a consistent state for $PID_{i}$ as in the registration algorithm; then it returns the full private key materials $\left(x_{i},ppk_{i}\right)$ honestly from $L_{ID}$.

(2) *AggVerify:* on inputting an aggregate candidate consisting of an aggregate scalar $\sigma_{agg}$ and a batch ${\left\{\left(m_{i},PID_{i},PK_{i},\pi_{i},T_{i},U_{i},\sigma_{i}\right)\right\}}_{i=1}^{n}$, $B_{3}$ samples a fresh coefficient vector $\eta =\{\eta_{2},\ldots ,\eta_{n}\}$ with $\eta_{i}\in \left[2,2^{\ell }\right]$ and sets $\eta_{1}=1$. It then runs *AggVerify* according to the protocol using this η and returns the decision bit. Let $Q_{av}$ denote the total number of such queries.

*Forgery:* After all the queries have been completed, $A_{III}$ outputs a valid aggregate forgery $\delta_{agg}^{*}$ on a batch ${\left\{\left(m_{i},PID_{i},PK_{i},\pi_{i},T_{i},\delta_{i}\right)\right\}}_{i=1}^{n}$, where $\delta_{i}=\left(\sigma_{i},U_{i}\right)$.

By the correctness of *AggVerify*, the acceptance of $\delta_{agg}^{*}$ under the sampled coefficients $\eta^{*}$ implies the weighted aggregate verification equation holds.

Moreover, since $\delta_{agg}^{*}$ is a Type-III forgery, there exists an index *k* such that the individual verification equation is not satisfied for the *k*-th constituent transcript.

For each constituent transcript $\delta_{i}=\left(\sigma_{i},U_{i}\right)$, define its verification residue as

$$\Delta_{i}≜\sigma_{i}\cdot P-\left(U_{i}+h_{3,i}\cdot \left(X_{i}+R_{i}\right)+h_{3,i}h_{1,i}\cdot P_{pub}\right),$$

Therefore, $\Delta_{k}\neq O$ for some index *k*. On the other hand, the weighted aggregate verification equation is equivalent to

$$\sum_{i=1}^{n}\eta_{i}^{*}\Delta_{i}=O.$$

For any fixed non-zero residue vector $\left(\Delta_{1},\ldots ,\Delta_{n}\right)$ with at least one $\Delta_{k}\neq O$, the simplified SET soundness guarantees that, over a uniformly sampled $\eta^{*}$ with $\eta_{i}^{*}\in \left[2,2^{\ell }\right]$, the probability that the above cancellation happens is at most $2^{-\ell }$. Therefore, each *AggVerify* attempt can succeed with probability at most $2^{-\ell }$, and by a union bound over at most $Q_{av}$ adaptive *AggVerify* queries and the final output, we have $\epsilon_{III}\leq \left(Q_{av}+1\right)\cdot 2^{-\ell }$, which is negligible for a proper choice of *ℓ*.

Therefore, the above proof shows that a successful Type-III information-injection forgery would require the invalid constituent transcripts to cancel out under the randomly sampled aggregation coefficients. By the soundness guarantee of the simplified SET, this event can occur only with negligible probability. Hence, such a Type-III forgery is infeasible for any PPT adversary, and the proposed scheme achieves aggregate soundness. □

### 7.2. Formal Verification by ProVerif

We use ProVerif to formally verify the protocol within the Dolev–Yao adversary model, in which the adversary controls the public channel and can eavesdrop on, replay and forge messages according to symbolic rules. Following the standard perfect-cryptography abstraction in ProVerif, the verification results complement our computational analysis, which is based on number-theoretic assumptions.

(1) We first declare the public/private constants, variables, channels, and cryptographic primitives, and introduce abstract interfaces for signature verification and RSA-accumulator membership proofs.

(2) We then model the protocol participants as processes and implement the full message flow of our scheme over a public channel, so that the adversary can intercept, modify, and forward all transmissions.

(3) Finally, we define session events and formulate secrecy and authentication queries. The secrecy targets include ID, wit, ppk, and *x*, while the authentication target is an injective correspondence between the user completion and verifier acceptance events.

**Figure 2:**
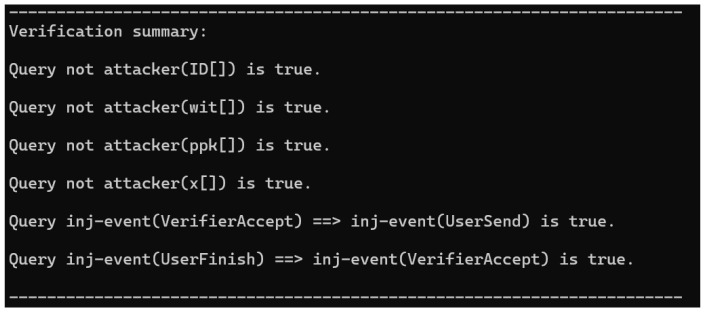
Verification results of our protocol through ProVerif.

As shown in Figure 2, all secrecy queries hold for ID, wit, ppk, and *x*, indicating that the network adversary cannot derive these values from protocol transcripts, thereby confirming the confidentiality of the protocol.

Moreover, ProVerif confirms the injective nature of our protocol’s authentication, ensuring that each verifier acceptance corresponds to a unique and genuine user execution. This demonstrates explicit resistance to replay attacks and provides consistent session matching for entity authentication.

### 7.3. Informal Security Analysis

To facilitate an intuitive understanding of the security rationale, we provide an informal analysis of the protocol construction. This explains how the desired security properties are achieved against network adversaries and malicious insiders.

(1) **Message authenticity and integrity:** a verifier only accepts a message if both the membership proof with respect to the latest accumulator value $acc_{j}$ and the signature verification succeed. Any modification to $\left(m_{i},PID_{i},PK_{i},T_{i}\right)$ changes the hash challenge and breaks verification, and an adversary without the legitimate signing secret cannot forge an authentication transcript that passes verification.

(2) **Anonymity and traceability:** the transmitted pseudonym $PID_{i}=\left(PID_{i,1},PID_{i,2}\right)$ together with the attached proof and signature does not reveal the real identity $ID_{i}$ to any entity on the public channel. Except for TA, any party can only validate the authentication without being able to recover $ID_{i}$; meanwhile, TA can extract the real identity from a reported transcript via $ID_{i}=PID_{i,2}⊕H_{0}\left(t\cdot X_{i},PID_{i,1}\right)$, thereby enabling traceability.

(3) **Revocability:** TA enforces revocation by updating the RSA accumulator, enabling a one-shot batch removal of all pseudonyms in the revoked user’s pseudonym pool so that the corresponding pseudonym primes $PID_{i,1}$ are no longer accumulated in the latest $acc_{j}$. Consequently, any authentication attempt initiated with any pseudonym of the revoked user will be rejected, and the revoked user cannot generate a valid membership proof for the current accumulator value.

(4) **Security properties of the NIZK proof:** the membership proof $\pi_{i}=\left(A_{i},C_{i}\right)$ is used to prove that the user holds a valid membership witness corresponding to the current RSA accumulator state, and it satisfies the following properties.

(i) Completeness: if ${\mathrm{User}}_{i}$ indeed holds a valid witness $wit_{i}$ and correctly generates $A_{i}$, $c_{i}$, and $C_{i}$ according to the protocol, the verification equation always holds, because $C_{i}^{PID_{i,1}}\equiv {\left(a_{i}\cdot wit_{i}^{c_{i}}\right)}^{PID_{i,1}}\equiv a_{i}^{PID_{i,1}}\cdot {\left(wit_{i}^{PID_{i,1}}\right)}^{c_{i}}\equiv A_{i}\cdot acc_{j}^{c_{i}}\;\left(\mathrm{mod}\;N\right)$.

(ii) Zero-knowledge: the publicly verifiable transcript contains only $\left(A_{i},C_{i}\right)$ and the challenge $c_{i}$ computed from public information, while the fresh randomness $a_{i}$ is re-sampled each time; thus, even for the same witness, many different transcripts can be produced, and no useful information about $wit_{i}$ is revealed.

(iii) Soundness: if an adversary does not hold a valid witness, producing an accepting $\left(A_{i},C_{i}\right)$ is equivalent to forging evidence that the membership relation holds without knowing $wit_{i}$. Under the Strong RSA assumption, such a forgery is infeasible.

(5) **Resistance to replay attacks:** each authentication transcript in the proposed scheme is associated with a timestamp $T_{i}$ and accepted only within the allowed freshness interval. Therefore, even if an adversary intercepts a valid transcript and replays it later, the verifier will reject it once the timestamp becomes outdated. In addition, the hash challenge depends on the transmitted message components and session-dependent inputs, so an old transcript cannot be reused in another authentication context without causing the verification equations to fail. Hence, the proposed scheme resists replay attacks.

(6) **Resistance to witness recovery attacks:** the user proves the membership relation $acc_{j}=wit_{i}^{PID_{i,1}}\;\mathrm{mod}\;N$ through a non-interactive zero-knowledge membership proof. Based on the completeness, zero-knowledge, and soundness properties of the above NIZK membership proof, the proof reveals no information about $wit_{i}$, and recovering $wit_{i}$ from the publicly verifiable proof transcript can be reduced to solving the Strong RSA problem over *N*. In addition, during membership updates, the TA broadcasts only product-form update exponents of pseudonym primes. Under the hardness assumption of integer factorization, even an insider adversary cannot determine which new pseudonyms have been added from such public broadcasts, let alone derive the corresponding witnesses. Therefore, the proposed scheme resists witness recovery attacks.

(7) **Resistance to identity grafting attacks:** in the proposed scheme, $PID_{i,1}$ is cryptographically bound to $PID_{i,2}$, $X_{i}$, and $R_{i}$ through the hash function, and this binding is further embedded into the partial private key $ppk_{i}$. Since $ppk_{i}$ is generated and distributed by the KGC, the user cannot arbitrarily modify this binding. As a result, the identity-related membership component and the signing-related key material cannot be recombined across different users. Even if an adversary obtains a valid membership component of another non-revoked pseudonym, it still cannot graft that component onto its own public key or partial private key to produce a valid authentication transcript. Hence, the proposed scheme resists identity grafting attacks.

(8) **Resistance to message linkability attacks:** the proposed scheme provides unlinkability by allowing each user to maintain a pseudonym pool and periodically switch the active pseudonym. Therefore, authentication transcripts generated in different sessions are associated with different pseudonyms, making it difficult for an external adversary to link them to the same user. Moreover, the real identity is concealed in the transmitted pseudonym, and only the TA can recover it when necessary. Hence, the proposed scheme resists message-linking attacks.

## 8. Performance Analysis

We evaluate the proposed scheme against other relevant CLS/CLAS schemes. Our analysis begins with a Security and Functionality Comparison, followed by a thorough evaluation of computation, communication, and revocation overheads. To ensure a fair and robust comparison, we select baselines that cover a range of cryptographic settings, including schemes based on bilinear mappings [15,27] and schemes that do not use bilinear mappings [8,19,21,30]. Furthermore, we benchmark the revocation functionality of our scheme against representative state-of-the-art schemes with user revocation support [25,27,28,30].

### 8.1. Security and Functionality Comparison

As summarized in Table 3, the compared schemes present different trade-offs in security and functionality. The schemes in [15,27] introduce heavyweight bilinear pairing operations. Moreover, several schemes do not achieve full certificateless security: refs. [19,21,25] cannot resist Type-I adversaries, and refs. [15,19,21,27] are not Type-III secure; refs. [30] is marked as “−” for Type-III since it does not explicitly define this notion. In addition, unlinkability is only supported by [25,30], while revocability is provided only by [25,27,28,30]. In contrast, our scheme is the only one that simultaneously achieves Type-I/II/III security, remains pairing-free, and supports both unlinkability and revocability, which better fits practical authentication in wireless medical sensor networks.

### 8.2. Computation Overhead Analysis

To provide a fair and reproducible evaluation of the performance discrepancies among different schemes under a consistent security level, we instantiate all primitives at the common 80-bit security threshold. Specifically, for ECC-based operations, we adopt a 160-bit elliptic curve, where the underlying prime field size is |p|=160 bits and the scalar field has |q|=160 bits, so elements in $Z_{q}^{*}$ are 20 bytes. Under the uncompressed representation, an elliptic-curve point is encoded as |G|=40 bytes. For pairing-based baselines, we instantiate pairing primitives on a Tate-pairing-friendly supersingular curve with embedding degree k=2; under the same setting, the prime field size is |p|=512 bits and a source-group element is encoded as $|G_{1}|=128$ bytes. For RSA-accumulator-related operations, we set the RSA modulus size to |N|=1024 bits to match the same 80-bit security level; thus, an element in $Z_{N}^{*}$ is encoded as 128 bytes.

We adopt the runtime benchmarks of basic cryptographic primitives reported in [8]. The measurements in [8] were obtained on an Intel Core i5-9500T 2.20 GHz platform with 8 GB RAM running Ubuntu 22.04, implemented in C/C++ using the MIRACL cryptographic library. Each primitive runtime is averaged over 1000 executions, as summarized in Table 4, and these benchmarks are used to translate the operation counts of SN/WN/MS into rough computation overhead in our evaluation.

**Figure 3:**
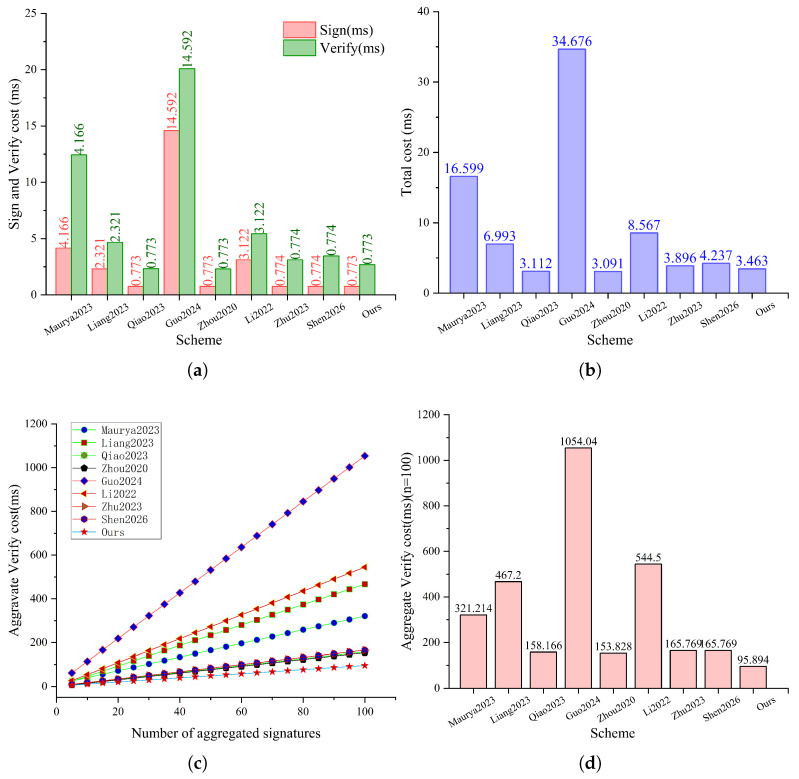
Computation overhead comparison. The panels are described as follows: (**a**) Individual signing and verification costs. (**b**) Total cost per authentication. (**c**) Aggregate verification cost versus the number of aggregated signatures *n*. (**d**) Aggregate verification cost comparison when n=100. Source: The data for Maurya2023, Liang2023, Qiao2023, Guo2024, Zhou2020, Li2022, Zhu2023, and Shen2026 are taken from [8,15,19,21,25,27,28,30], respectively.

For the computation overhead, we focus on the costs of individual signing/verification and aggregate verification. The operation counts and the derived time expressions are summarized in Table 5, and the results are illustrated in Figure 3.

As shown in Figure 3a,b, our scheme maintains a low individual cost. Individual signing requires $T_{em}+T_{h}$ and individual verification requires $3T_{em}+3T_{ea}+3T_{h}+2T_{me}$, resulting in a total cost of 3.463 ms per authentication. It is clearly more efficient than pairing-based schemes [15,27] since their runtime is dominated by heavyweight pairing-related operations. Compared with pairing-free designs [19,25], our total cost per authentication is slightly higher; however, Table 3 shows that refs. [19,25] do not achieve full security and functionality simultaneously, where ref. [19] fails to resist Type-I adversaries and does not provide unlinkability or revocability, and ref. [25] cannot resist Type-I adversaries.

For aggregate verification, the overhead of all schemes increases linearly with the number of aggregated signatures *n* as depicted in Figure 3c. Our aggregate verification cost is $\left(n+2\right)T_{em}+3nT_{ea}+\left(2n-2\right)T_{sem}+2nT_{h}=0.945n+1.394$, which yields the lowest growth rate among the compared schemes; for instance, when n=100, our scheme requires 95.894 ms as shown in Figure 3d.

Overall, the above results demonstrate that our scheme is computationally efficient, offering low individual authentication costs and a small, linear growth rate in aggregate verification as *n* increases.

### 8.3. Communication Overhead Analysis

**Figure 4:**
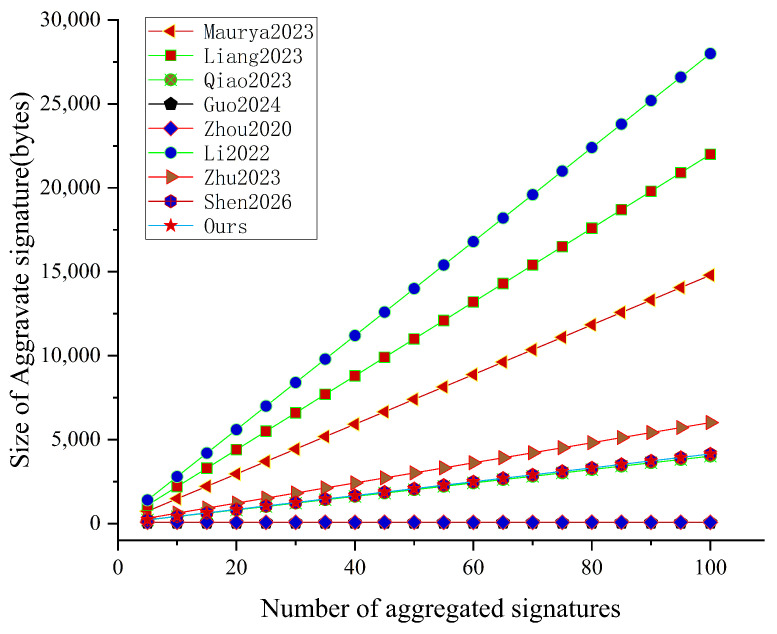
Aggregate signature size versus the number of aggregated signatures n (l = 10). Source: The data for Maurya2023, Liang2023, Qiao2023, Guo2024, Zhou2020, Li2022, Zhu2023, and Shen2026 are taken from [8,15,19,21,25,27,28,30], respectively.

For the communication overhead, we compare the related schemes in terms of the transmitted signature length. Let $|G_{1}|$, |G|, and $|Z_{q}^{*}|$ denote the element sizes in $G_{1}$, G, and $Z_{q}^{*}$, respectively. Under the 80-bit security setting, we set $|G_{1}|=128$ bytes, |G|=40 bytes, and $|Z_{q}^{*}|=20$ bytes. The signature-size comparison results are summarized in Table 6, and the aggregate signature size versus the number of aggregated signatures *n* is shown in Figure 4.

In our scheme, the size of an individual signature is 60 bytes, which is the smallest among the schemes compared in Table 6, indicating a lightweight per-message transmission cost for frequent authentications. For the aggregate signature, in addition to the *n* group elements, our construction introduces a compact extra term derived from the small-integer masking vector, where multiple small integers are efficiently packed into $Z_{q}^{*}$ elements. Let *l* denote the bit-length of each small integer and let *A* denote the bit-length budget associated with the target security level; then the aggregate signature size is $n|G|+\left(\left\lceil nl/(2A)\right\rceil +1\right)\left|Z_{q}^{*}\right|$. As illustrated in Figure 4, the aggregate signature size grows linearly with *n*, and our scheme shares the same low growth rate as [8], remaining in a relatively small range even when a large number of signatures are aggregated. In contrast, several schemes incur a much steeper linear growth due to transmitting multiple group elements and/or multiple $Z_{q}^{*}$ elements per signer, resulting in larger aggregated messages. Although [25,27] achieve constant-size aggregate signatures, they cannot provide comparable security and functionality simultaneously: ref. [25] fails to resist Type-I adversaries, and ref. [27] relies on heavyweight bilinear pairing operations and does not provide unlinkability.

Therefore, our scheme achieves favorable communication efficiency. The individual signature is compact, and the aggregate signature size grows linearly with *n*, which has a relatively low growth rate compared to other schemes.

### 8.4. Revocation Overhead Analysis

**Figure 5:**
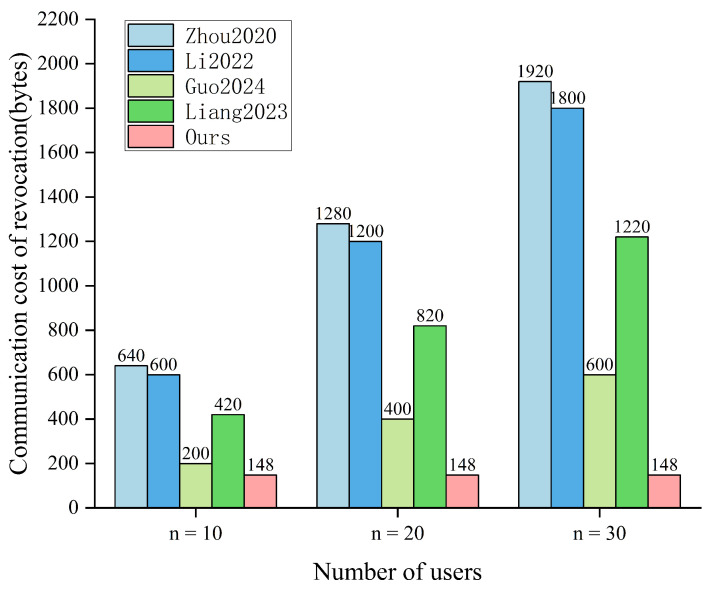
Revocation communication costs. Source: The data for Zhou2020, Li2022, Guo2024 and Liang2023 are taken from [25,27,28,30], respectively.

To evaluate the extra burden introduced by revocation, we compare our scheme with representative revocable schemes from two aspects, namely, revocation communication overhead and revocation storage overhead. Following the same byte-length setting as in Section 8.3, we set $|G_{1}|=128$ bytes, |G|=40 bytes, and $|Z_{q}^{*}|=20$ bytes; to quantify accumulator-related update information, we set $|Z_{N}^{*}|=128$ bytes. A pseudonym-related identifier is treated as a fixed-length bitstring and counted as 20 bytes, which can be encoded as one element in $Z_{q}^{*}$, and the timestamp $t_{i}$ is assumed to be 4 bytes. In the revocation communication comparison, we only count the revocation-specific update payload delivered from TA to users and omit auxiliary authentication fields used solely to authenticate the update message such as broadcast signatures, and their verification-related components, because these fields can be instantiated in the same manner across schemes and do not affect the underlying revocation mechanism. Moreover, we consider the overhead incurred when revoking a single user while the system still contains *n* active users. The comparison results are summarized in Table 7, and Figure 5 shows the revocation communication costs under different revocation schemes.

In terms of revocation communication, the schemes in [25,27,28] essentially rely on point-to-point dissemination. The manager needs to deliver revocation-related information to each affected user, so the communication cost increases linearly with the number of users. Specifically, refs. [27,28] use time-periodic key updates, and revocation effectiveness usually depends on the next update cycle. This can result in delayed membership changes. In contrast, ref. [30] adopts revocation by broadcast polynomial for revocation, which avoids per-user delivery. However, the broadcast payload still increases linearly with system scale, as the coefficient set $\left\{B_{0},a_{0},\ldots ,a_{n}\right\}$ expands with *n*. In our scheme, only the set of values $\left\{acc_{new},E\right\}$ is broadcast per revocation event. Thus, the revocation communication overhead remains constant at O(1), regardless of *n*. This advantage is clearly reflected in Figure 5, where the cost of our scheme remains at 148 bytes, whereas the cost of other schemes increases with *n*.

In terms of revocation storage, ref. [25] needs to maintain an explicit revocation list ($\left\{FID_{1},\ldots ,FID_{n}\right\}$), which results in linear storage growth as revoked users accumulate. The time-periodic approaches in [27,28] also require storing per-user records or update materials, leading to storage overhead proportional to *n*. Although ref. [30] only stores a compact value *B* and thus achieves constant storage, its revocation broadcast message still grows with *n*, making it difficult to simultaneously achieve scalable communication and storage. Benefiting from the RSA accumulator, our scheme only keeps the latest accumulator value $acc_{new}$, which restricts the revocation storage overhead to O(1) and avoids long-term linear expansion.

Overall, our scheme achieves a constant overhead size for both revocation communication and storage. This makes it well-suited for large-scale wireless medical sensor networks deployments with frequent membership changes.

## 9. Discussion

Although the proposed scheme achieves the intended security and privacy objectives under the adopted threat model, practical deployment in wireless medical sensor networks still involves system-level considerations beyond formal security proofs and theoretical cost analysis. This section discusses the engineering feasibility of the newly introduced computations on resource-constrained sensor nodes, summarizes the main limitations of the current approach, and outlines directions for future improvements and extensions.

### 9.1. Implementation Feasibility

Our enhanced design introduces several mechanisms to strengthen security and privacy. Among them, the components that are most relevant to the implementation burden on end devices mainly include: the added non-interactive zero-knowledge (NIZK) membership proof in the authentication phase, and the dynamic pseudonym pool with periodic rotation for achieving unlinkability. Accordingly, this subsection focuses on (1) the feasibility of ZK proof generation on resource-constrained sensor nodes and (2) the management of dynamic pseudonym pools and the associated overhead trade-offs.

First, regarding ZK membership proof generation, we adopt a Fiat–Shamir-based non-interactive membership proof, whose computation can be naturally decomposed into offline precomputation and lightweight online generation. Specifically, the randomness and intermediate values related to the commitment can be precomputed and cached when the node is idle; in the online phase, the node only needs to derive the hash challenge from the current message and context and then perform a small number of scalar operations to output the proof. As a result, the major cost of the added proof step can be shifted to the offline stage, keeping the extra overhead on the real-time authentication path low. Moreover, the required basic primitives, such as hashing and modular exponentiation, are consistent with those used elsewhere in the protocol, facilitating the reuse of existing cryptographic libraries on embedded platforms.

Second, regarding the management of the dynamic pseudonym pool, the pool size *n* is a configurable parameter that balances unlinkability and resource consumption: a larger *n* reduces the reuse frequency of any single pseudonym and hence enhances long-term unlinkability; however, the node must store *n* tuples $\left(PID_{k},PK_{k},wit_{k}\right)$ and the associated secret materials, and it must also update the witnesses for all stored pseudonyms upon membership updates, leading to storage and maintenance costs that grow linearly with *n*. It is worth emphasizing that each authentication session uses only one pseudonym, so the per-session signing and verification costs are essentially independent of *n*; the system sensitivity to *n* mainly stems from the combined effect of pool size and update frequency. Considering the resource constraints of WMSN devices and the dynamics of hospital scenarios, a moderate *n* can be selected according to the storage budget and the expected update frequency. In addition, two engineering strategies can be adopted: (i) a threshold replenishment mechanism for pseudonym resources, where the node requests and loads a new batch of pseudonyms from the TA once the number of remaining unused pseudonyms falls below a preset threshold to avoid pool depletion and sustain rotation; and (ii) a batched, on-demand witness refresh strategy, which amortizes the update computation over multiple reporting periods. These strategies improve practicality without changing the protocol logic, while preserving unlinkability and feasibility.

### 9.2. Limitations

This scheme relies upon a Trusted Authority (TA) as a fully trustworthy and perpetually available authority entity, responsible for issuing pseudonym pools, maintaining RSA accumulator states, and broadcasting membership updates. In practical deployment, the TA may present a single point of failure: should the TA be compromised or temporarily unavailable, the security of witness issuance and the continuity of dynamic membership management would be jeopardized.

Although our performance evaluation is systematic, it primarily relies on theoretical performance analysis and formal simulation verification. End-to-end testing has not yet been conducted in real-world WMSNs scenarios or on actual sensor hardware platforms. Considering that real wireless medical environments often exhibit link quality fluctuations, interference, and packet loss and retransmission issues, the actual runtime latency and load of the protocol may deviate from theoretical estimates.

The current design prioritizes authentication and signature functions to guarantee integrity, confidentiality, and revocability but does not provide an end-to-end encryption mechanism to safeguard the confidentiality of medical payloads.

### 9.3. Future Work

In the future, we plan to develop a system prototype and construct a more realistic wireless medical sensor network (WMSN) test platform for end-to-end evaluation. This will include assessing overall authentication latency, actual energy consumption, and robustness under unstable wireless channels, thereby further validating the engineering feasibility of the proposed protocol in complex medical environments.

To address potential single-point-of-failure risks in the TA, we will explore more robust distributed alternatives, such as blockchain-based distributed ledgers or secret sharing techniques, to support credential issuance and revocation management. This will enhance system availability and mitigate the impact of TA compromise.

We will investigate integrating lightweight cryptographic mechanisms at the protocol layer to provide end-to-end confidentiality for medical payloads, further strengthening security and privacy protection over open wireless links.

Future work will introduce data aggregation mechanisms to further reduce bandwidth consumption and improve overall efficiency. Concurrently, we will study system-level design considerations and challenges when combining data aggregation with signature aggregation in wireless medical sensor networks.

## 10. Conclusions

This paper analyzes the recently proposed revocable certificate-free aggregate authentication scheme based on RSA accumulators by Shen et al. [8], revealing its potential security vulnerabilities and presenting concrete attack scenarios. Building upon this foundation, we propose a security-enhanced pairing-free certificateless aggregate authentication protocol with efficient revocation. This protocol integrates strong identity–membership binding, non-interactive zero-knowledge membership proofs, and a dynamic pseudonym rotation mechanism. It satisfies the requirements for lightweight authentication, privacy protection, and dynamic membership management in large-scale resource-constrained deployment environments. Formal analysis and performance comparisons demonstrate that, at equivalent security levels, this protocol achieves compact communication overhead and efficient aggregate verification while maintaining constant revocation update overhead. It is thus suitable for large-scale WMSNs deployments with frequent member changes.

## Figures and Tables

**Table 1:** Notations in our proposal.

Symbol	Definition
λ	Security parameter
*t*	Private key of TA
$T_{pub}$	Public key of TA
*s*	Private key of KGC
$P_{pub}$	Public key of KGC
params	System public parameters
$ID_{i}$	Real identity of $SN_{i}$
$PID_{i}$	Pseudonym of $SN_{i}$
$ID_{prime}$	Prime selected for pseudonym binding
$ppk_{i}$	Partial private key of $SN_{i}$
$sk_{i}$	Full private key of $SN_{i}$
$PK_{i}$	Public key of $SN_{i}$
acc	RSA accumulator value
$wit_{i}$	Membership witness of $SN_{i}$
$\pi_{i}$	NIZK membership proof
$\delta_{i}$	Authentication signature of $SN_{i}$
$m_{i}$	Medical message
$T_{i}$	Timestamp
AUX	Accumulator update message

**Table 2:** Summary of key improvements on Shen et al.’s [8] scheme.

Aspect	Limitations in Shen et al.’s [8]	Improvements in Ours
**Security**	Vulnerable to witness recovery attack.	Non-interactive zero-knowledge proof.
**Revocation**	Vulnerable to identity grafting attack.	Strong identity–membership binding.
**Unlinkability**	Static single pseudonym.	Pseudonym pool with dynamic rotation.
**Complexity**	Multi-hash-assisted binding structure.	Single-hash-driven signature design.

**Table 3:** Security and functionality comparison with related schemes.

Property	[15]	[30]	[19]	[27]	[25]	[28]	[21]	[8]	Ours
**Resistance to Type-I**	✓	✓	×	✓	×	✓	×	✓	✓
**Resistance to Type-II**	✓	✓	✓	✓	✓	✓	✓	✓	✓
**Resistance to Type-III**	×	−	×	×	✓	✓	×	✓	✓
**Without Pairing**	×	✓	✓	×	✓	✓	✓	✓	✓
**Unlinkability**	×	✓	×	×	✓	×	×	×	✓
**Revocability**	×	✓	×	✓	✓	✓	×	×	✓

“−” indicates that the corresponding security notion is not explicitly defined in the original scheme.

**Table 4:** Execution time of cryptographic operations.

Symbol	Meaning	Time (ms)
$T_{bp}$	Bilinear Pairing	2.412
$T_{bpm}$	Pairing-based Scalar Multiplication	1.039
$T_{bpa}$	Pairing-based Point Addition	0.008
$T_{bhtp}$	Pairing-based Map-to-point Hash	2.607
$T_{em}$	Scalar Multiplication	0.772
$T_{sem}$	Small Scalar Multiplication	0.075
$T_{ea}$	Point Addition	0.007
$T_{h}$	General Secure Hash	0.001
$T_{me}$	Modular Exponentiation	0.175

**Table 5:** Computation overhead comparison among related schemes.

Scheme	Individual Signing (ms)	Individual Verification (ms)	Aggregate Verification (ms)
[15]	$4T_{bpm}+T_{bpa}+2T_{h}$	$3T_{bp}+5T_{bpm}+2T_{h}$	$3T_{bp}+\left(3n+2\right)T_{bpm}+2nT_{h}=3.119n+9.314$
[30]	$3T_{em}+5T_{h}$	$6T_{em}+5T_{ea}+5T_{h}$	$6nT_{em}+5nT_{ea}+5nT_{h}=4.672n$
[19]	$T_{em}+T_{h}$	$3T_{em}+3T_{ea}+2T_{h}$	$\left(2n+1\right)T_{em}+\left(4n-1\right)T_{ea}+\left(2n+1\right)T_{h}=1.574n+0.766$
[25]	$T_{em}+T_{h}$	$3T_{em}+2T_{h}$	$\left(2n-1\right)T_{em}+2nT_{h}=1.546n-0.772$
[27]	$4T_{bpm}+T_{bpa}+4T_{bhtp}$	$4T_{bp}+T_{bpa}+4T_{bhtp}$	$4T_{bp}+\left(2n-1\right)T_{bpa}+4nT_{bhtp}=10.444n+9.640$
[28]	$4T_{em}+4T_{ea}+6T_{h}$	$7T_{em}+5T_{ea}+6T_{h}$	$7nT_{em}+5nT_{ea}+6nT_{h}=5.445n$
[21]	$T_{em}+2T_{h}$	$4T_{em}+3T_{ea}+3T_{h}$	$\left(2n+2\right)T_{em}+3nT_{ea}+\left(n-1\right)T_{sem}+3nT_{h}=1.643n+1.469$
[8]	$T_{em}+2T_{h}$	$4T_{em}+3T_{ea}+4T_{h}+2T_{me}$	$\left(2n+2\right)T_{em}+3nT_{ea}+\left(n-1\right)T_{sem}+3nT_{h}=1.643n+1.469$
Ours	$T_{em}+T_{h}$	$3T_{em}+3T_{ea}+3T_{h}+2T_{me}$	$\left(n+2\right)T_{em}+3nT_{ea}+\left(2n-2\right)T_{sem}+2nT_{h}=0.945n+1.394$

**Table 6:** Communication overhead comparison in terms of signature size (|Λ| = 80, l = 10).

Scheme	Individual Signature (Bytes)	Aggregate Signature (Bytes)
[15]	$G_{1}+Z_{q}^{*}=148$	$nG_{1}+nZ_{q}^{*}=148n$
[30]	$4G+3Z_{q}^{*}=220$	$4nG+3nZ_{q}^{*}=220n$
[19]	$G+Z_{q}^{*}=60$	$nG+Z_{q}^{*}=40n+20$
[25]	2G=80	2G=80
[27]	$G+Z_{q}^{*}=60$	$G+Z_{q}^{*}=60$
[28]	$6G+2Z_{q}^{*}=280$	$6nG+2nZ_{q}^{*}=280n$
[21]	$G+Z_{q}^{*}=60$	$nG+\left(n+1\right)Z_{q}^{*}=60n+20$
[8]	$G+Z_{q}^{*}=60$	$nG+\left(nl/2|\Lambda |+1\right)Z_{q}^{*}=41.25n+20$
**Ours**	$G+Z_{q}^{*}=60$	$nG+\left(nl/2|\Lambda |+1\right)Z_{q}^{*}=41.25n+20$

**Table 7:** Revocation overhead comparison in terms of communication and storage.

Scheme	Communication Message	Overhead	Size (Bytes)	Storage Data	Overhead	Size (Bytes)
[27]	$\left\{ID_{i},T_{i},t_{i}\right\}$	$n\left(\left|G|+\right|Z_{q}^{*}\left|+|T\right|\right)$	64n	$n\cdot \{ID_{i},T_{i},t_{i}\}$	$n\left(\left|G|+\right|Z_{q}^{*}\left|+|T\right|\right)$	64n
[28]	$\left\{\Upsilon_{AID_{i}},\xi_{AID_{i},t}\right\}$	$n\left(\left|G|+\right|Z_{q}^{*}|\right)$	60n	$n\cdot \{AID_{i},t_{i}\}$	$n\left(|Z_{q}^{*}\left|+|T\right|\right)$	24n
[25]	$\left\{FID_{i}\right\}$	$n|Z_{q}^{*}|$	20n	$n\{FID_{i}\}$	$n|Z_{q}^{*}|$	20n
[30]	$\left\{B_{0},a_{0},a_{1},\ldots ,a_{n}\right\}$	$\left|G|+n\right|Z_{q}^{*}|$	40+20n	*B*	$|Z_{q}^{*}|$	20
Ours	$\left\{acc_{new},E\right\}$	$|Z_{N}^{*}\left|+\right|Z_{q}^{*}|$	148	$acc_{new}$	$|Z_{N}^{*}|$	128

## Data Availability

All data will be provided upon request to the authors.

## References

[1] Zhu F., Yi X., Abuadbba A., Khalil I., Nepal S., Huang X., Yan X. (2021). Certificate-based anonymous authentication with efficient aggregation for wireless medical sensor networks. IEEE Internet Things J..

[2] Chen X., Hu C., Chen Y., Xia X., Cai B., Yu J. (2026). An enhanced security data transmission scheme for wireless medical sensor network. J. Mach. Learn. Inf. Secur..

[3] Kumar P., Lee H.-J. (2011). Security issues in healthcare applications using wireless medical sensor networks: A survey. Sensors.

[4] Sangari A.S., Manickam J.M.L. (2014). Public key cryptosystem based security in wireless body area network. Proceedings of the 2014 International Conference on Circuits, Power and Computing Technologies (ICCPCT-2014).

[5] Ding R., Zhong H., Ma J., Liu X., Ning J. (2019). Lightweight privacy-preserving identity-based verifiable IoT-based health storage system. IEEE Internet Things J..

[6] Liu Y., He Z., Liang J., Li Z., Deng Q. (2026). Multidimensional trust evaluation and task match based workers recruitment scheme for MCS. IEEE Trans. Dependable Secure Comput..

[7] Zhao Y., Hou Y., Wang L., Kumari S., Khan M.K., Xiong H. (2020). An efficient certificateless aggregate signature scheme for the Internet of Vehicles. Trans. Emerg. Telecommun. Technol..

[8] Shen Z., Kou X., Yang T. (2026). An efficient certificateless authentication scheme based on RSA accumulator for smart healthcare. J. Inf. Secur. Appl..

[9] Kaur R., Shahrestani S., Ruan C. (2024). Security and privacy of wearable wireless sensors in healthcare: A systematic review. Comput. Netw. Commun..

[10] Siddiqui Z., Gao J., Khan M.K. (2022). An improved lightweight PUF–PKI digital certificate authentication scheme for the Internet of Things. IEEE Internet Things J..

[11] Shamir A. (1984). Identity-based cryptosystems and signature schemes. Proceedings of the Workshop on the Theory and Application of Cryptographic Techniques.

[12] Sharma G., Bala S., Verma A.K. (2017). PF-IBS: Pairing-free identity based digital signature algorithm for wireless sensor networks. Wirel. Pers. Commun..

[13] Al-Riyami S.S., Paterson K.G. (2003). Certificateless public key cryptography. Proceedings of the International Conference on the Theory and Application of Cryptology and Information Security.

[14] Yang Y., Zhang L., Zhao Y., Choo K.-K.R., Zhang Y. (2022). Privacy-preserving aggregation-authentication scheme for safety warning system in fog-cloud based VANET. IEEE Trans. Inf. Forensics Secur..

[15] Maurya C., Chaurasiya V.K. (2023). Efficient anonymous batch authentication scheme with conditional privacy in the Internet of Vehicles (IoV) applications. IEEE Trans. Intell. Transp. Syst..

[16] Meher B.K., Amin R., Abdussami M., Sureshkumar V., Hossain M.A. (2024). Efficient certificateless anonymous mutual authentication in WBANs for smart healthcare. IEEE Trans. Intell. Transp. Syst..

[17] Gayathri N.B., Thumbur G., Kumar P.R., Rahman M.Z.U., Reddy P.V., Lay-Ekuakille A. (2019). Efficient and secure pairing-free certificateless aggregate signature scheme for healthcare wireless medical sensor networks. IEEE Internet Things J..

[18] Liu J., Wang L., Yu Y. (2020). Improved security of a pairing-free certificateless aggregate signature in healthcare wireless medical sensor networks. IEEE Internet Things J..

[19] Qiao Z., Yang Q., Zhou Y., Yang B., Zhang M. (2023). A novel construction of certificateless aggregate signature scheme for healthcare wireless medical sensor networks. Comput. J..

[20] Yan Z., Qu H., Lin X.-J. (2024). On the security of a novel construction of certificateless aggregate signature scheme for healthcare wireless medical sensor networks. Comput. J..

[21] Zhu F., Yi X., Abuadbba A., Khalil I., Huang X., Xu F. (2023). A security-enhanced certificateless conditional privacy-preserving authentication scheme for vehicular ad hoc networks. IEEE Trans. Intell. Transp. Syst..

[22] Yang X., Li S., Yang L., Du X., Wang C. (2024). Efficient and security-enhanced certificateless aggregate signature-based authentication scheme with conditional privacy preservation for VANETs. IEEE Trans. Intell. Transp. Syst..

[23] Wu W., Heng Y. (2025). An efficient certificateless aggregate signature scheme resistant to collusion attacks for VANETs. Comput. Netw..

[24] Zhang J., Zhong H., Cui J., Xu Y., Liu L. (2020). An extensible and effective anonymous batch authentication scheme for smart vehicular networks. IEEE Internet Things J..

[25] Guo R., Dong R., Li X., Zhang Y., Zheng D. (2024). DRCLAS: An efficient certificateless aggregate signature scheme with dynamic revocation in vehicular ad-hoc networks. Veh. Commun..

[26] Zhang K., Xue Z., Li S., Deng Y., Liu Z. (2025). An Efficient and Security-Enhanced Certificateless Aggregate Signature for VANETs. Proceedings of the 2025 10th International Conference on Intelligent Computing and Signal Processing (ICSP).

[27] Zhou F., Li Y., Lin C. (2020). A revocable certificateless aggregate signature scheme with enhanced security. Int. J. Netw. Secur..

[28] Li X., Jiang C., Du D., Fei M., Wu L. (2022). A novel revocable lightweight authentication scheme for resource-constrained devices in cyber–physical power systems. IEEE Internet Things J..

[29] Wang Y., Liu Y., Tian Y. (2022). ISC-CPPA: Improverd-security certificateless conditional privacy-preserving authentication scheme with revocation. IEEE Trans. Veh. Technol..

[30] Liang Y., Yan H., Liu Y. (2023). Unlinkable signcryption scheme for multi-receiver in VANETs. IEEE Trans. Intell. Transp. Syst..

[31] Al-Mekhlafi Z.G., Al-Janabi H.D.K., Al-Shareeda M.A., Mohammed B.A., Alshudukhi J.S., Al-Dhlan K.A. (2024). Fog computing and blockchain technology based certificateless authentication scheme in 5G-assisted vehicular communication. Peer-to-Peer Netw. Appl..

[32] Camacho P., Hevia A., Kiwi M., Opazo R. (2008). Strong accumulators from collision-resistant hashing. Proceedings of the International Conference on Information Security.

[33] Camenisch J., Kohlweiss M., Soriente C. (2009). An accumulator based on bilinear maps and efficient revocation for anonymous credentials. Proceedings of the International Workshop on Public Key Cryptography.

[34] Li Y., Cao L., Zheng G., Men H., Chen L. (2024). Improved RSA dynamic cryptographic accumulator-based anonymous batch authentication scheme for Internet of Vehicles. Comput. Electr. Eng..

[35] Fiat A., Shamir A. (1986). How to prove yourself: Practical solutions to identification and signature problems. Proceedings of the Conference on the Theory and Application of Cryptographic Techniques.

[36] Nymann J.E. (1972). On the probability that k positive integers are relatively prime. J. Number Theory.

[37] Bellare M., Neven G. (2006). Multi-signatures in the plain public-key model and a general forking lemma. Proceedings of the 13th ACM Conference on Computer and Communications Security.

[38] Hwang J.Y., Song B., Choi D., Jin S.-H., Cho H.S., Lee M.-K. (2017). Simplified small exponent test for batch verification. Theor. Comput. Sci..

